# Endogenous RALF peptide function is required for powdery mildew host colonization

**DOI:** 10.1111/nph.71282

**Published:** 2026-05-27

**Authors:** Henriette Leicher, Sebastian D. Schade, Jan W. Huebbers, Kristina S. Munzert‐Eberlein, Genc Haljiti, David Biermann, Athanasios Makris, Xiaoxuan Zhu, Yashank Chauhan, Christina Ludwig, Marion C. Müller, Toshinori Kinoshita, Timo Engelsdorf, Julien Gronnier, Martina K. Ried‐Lasi, Aurélien Boisson‐Dernier, Ralph Hückelhoven, Martin Stegmann

**Affiliations:** ^1^ Phytopathology, TUM School of Life Sciences Technical University of Munich (TUM) 85354 Freising Germany; ^2^ Unit of Plant Molecular Cell Biology, Institute for Biology I RWTH Aachen University 52056 Aachen Germany; ^3^ Molecular Plant Physiology, Department of Biology Philipps‐Universität Marburg 35043 Marburg Germany; ^4^ Bavarian Center for Biomolecular Mass Spectrometry (BayBioMS), TUM School of Life Science Technical University of Munich (TUM) 85354 Freising Germany; ^5^ Plant Cell Biology, TUM School of Life Sciences Technical University of Munich (TUM) 85354 Freising Germany; ^6^ Leibniz‐Institute of Plant Biochemistry 06120 Halle Germany; ^7^ Université Côte d'Azur INRAE, Institut Sophia Agrobiotech 06903 Sophia Antipolis Cedex France; ^8^ Graduate School of Science Nagoya University Chikusa 464‐8602 Nagoya Japan; ^9^ Institute of Transformative Bio‐Molecules (WPI‐ITbM) Nagoya University Chikusa 464‐8602 Nagoya Japan; ^10^ Present address: Institute of Botany, Molecular Botany Ulm University 89081 Ulm Germany

**Keywords:** apoplastic pH homeostasis, cell wall, powdery mildew, RALF peptides, receptor kinases, susceptibility factor

## Abstract

- The receptor kinase FERONIA (FER) is a susceptibility factor for biotrophic powdery mildew fungal pathogens in *Arabidopsis thaliana*, but the underlying molecular mechanisms remain largely unknown. FER is required for the perception of endogenous RAPID ALKALINIZATION FACTOR (RALF) peptides to control various aspects of plant growth, development and immunity. RALFs are either perceived by FER/LORELEI‐LIKE GPI‐ANCHORED PROTEIN (LLG) heterocomplexes to induce cellular responses or bind to LEUCINE‐RICH REPEAT EXTENSIN (LRX) proteins as cell wall structural components.
- Combining genetics, cell biology and biochemistry, we found that FER's endogenous RALF ligands are necessary for full colonization success of the powdery mildew‐species *Erysiphe cruciferarum*. We reveal that LLGs and LRXs are also powdery mildew susceptibility factors. We show that cell wall remodeling and apoplastic pH homeostasis, hallmark features of RALF function, support powdery mildew reproductive success. We provide data that RALF‐dependent powdery mildew pathogenesis is partially independent of FER.
- Powdery mildew fungi likely do not produce RALF peptide mimics, suggesting their reliance on endogenous RALFs for successful host colonization.
- We propose that powdery mildew fungi require RALF‐mediated modulation of apoplastic pH and pectin remodeling for successful host colonization, highlighting a new susceptibility mechanism by obligate biotrophic fungi.

## Graphical abstract

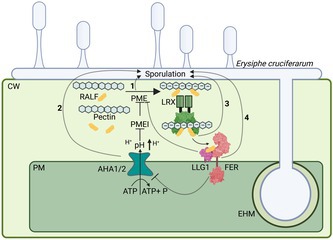

## Introduction

Powdery mildew is one of the most widespread fungal plant diseases affecting several economically relevant crop species (Glawe, 2008), with infection being characterized by continuous interaction between the biotrophic fungus and its host plant. Susceptibility genes (S‐genes) can affect fungal infection success and are utilized by pathogens for their own benefit. In consequence, mutating these genes usually confers pathogen and genotype‐independent and hence durable disease resistance (van Schie & Takken, 2014; Engelhardt *et al*., 2018). The first well‐characterized powdery mildew susceptibility factor was the barley MILDEW LOCUS O (MLO) that confers barley susceptibility toward adapted *Blumeria hordei*. Mutants in orthologous *MLO* genes are powdery mildew resistant in phylogenetically diverse plant species, including *Arabidopsis thaliana* (hereafter Arabidopsis) and tomato (Kusch & Panstruga, 2017).

Next to susceptibility factors, the outcome of plant–pathogen interactions is shaped by the plant immune system. Pattern‐triggered immunity (PTI) is the first line of induced resistance and activated by the perception of microbe‐associated molecular patterns (MAMPs) by pattern recognition receptors (PRRs), which are often plasma membrane‐localized receptor‐like kinases (RLKs). To subvert PTI, adapted pathogens evolved effectors that are mainly perceived by intracellular nucleotide‐binding leucine‐rich repeat receptors (NB‐LRRs), activating pathogen race‐specific effector‐triggered immunity (Ngou *et al*., 2022).

In addition to their function as PRRs, RLKs are important sensors for endogenous signals regulating growth, abiotic stress and/or modulating immunity (Rzemieniewski & Stegmann, 2022; Zecua‐Ramirez *et al*., 2026). Adapted pathogens can hijack RLK pathways to support accommodation (Hok *et al*., 2011, 2014; Ried *et al*., 2019). The conserved RLK FERONIA (FER) and related CATHARANTUS ROSEUS RECEPTOR‐LIKE KINASE 1‐LIKEs (CrRLK1Ls) perceive endogenous RAPID ALKALINIZATION FACTOR (RALF) peptides to regulate multiple plant physiological processes (Ge *et al*., 2017; Gonneau *et al*., 2018; Zhu *et al*., 2021; Zhong *et al*., 2022; Lan *et al*., 2023). FER recognizes RALFs in a heteromeric complex with LORELEI‐LIKE GPI‐ANCHORED PROTEIN 1 (LLG1; Li *et al*., 2015; Xiao *et al*., 2019) and controls PTI as a RALF‐regulated scaffold for PRR complexes and their nanoscale organization (Stegmann *et al*., 2017; Gronnier *et al*., 2022). In addition to FER‐dependent signaling, recent reports show that RALFs have a structural role in organizing the patterning of demethylesterified pectins in the cell wall (Moussu *et al*., 2023; Schoenaers *et al*., 2024). Here, RALFs bind to cell wall bound LEUCINE‐RICH REPEAT EXTENSIN (LRX) proteins but the biochemical interplay between LRX and FER‐LLG complexes remains little understood (Mecchia *et al*., 2017; Zhao *et al*., 2018; Dünser *et al*., 2019; Moussu *et al*., 2020; Gronnier *et al*., 2022; Schade *et al*., 2025). Adapted pathogens can hijack RALF signaling for their own benefit. *Fusarium oxysporum* and root knot nematodes produce RALF mimics to enhance infection success on tomato and Arabidopsis (Masachis *et al*., 2016; Thynne *et al*., 2017; Zhang *et al*., 2020). Furthermore, the Ler‐0 *fer‐1* mutant is more resistant to the powdery mildew‐species *Golovinomyces orontii*, suggesting that FER acts as an *S*‐factor (Kessler *et al*., 2010). Yet, how FER confers powdery mildew susceptibility remains largely unknown.

Here, we investigated the molecular mechanism of FER‐mediated powdery mildew susceptibility, using the Arabidopsis‐adapted powdery mildew fungus *Erysiphe cruciferarum* (*Ecr*). We show that LLG1 and LRX1‐LRX5 are required for successful powdery mildew infection. By loss of function studies, we identify RALF peptides as important susceptibility factors for powdery mildew. CRISPR Cas9‐generated *ralf22/23/33* (CRISPR *ralf3x*) and *ralf1/18/22/23/31/33/34* (CRISPR *ralf7x*) mutants show increasing resistance to *Ecr* infection depending on the number of eliminated RALFs. Our bioinformatic analysis indicates that powdery mildew fungi do not encode functional RALF peptide mimics, supporting that they require host‐endogenous RALFs for their own benefit (Masachis *et al*., 2016; Thynne *et al*., 2017). We suggest that the enhanced resistance of *fer* and CRISPR *ralf7x* is not associated with defects in MLO‐mediated penetration, or deregulation of defense‐related phytohormone signaling. Instead, powdery mildew might require FER‐RALF‐mediated apoplastic pH regulation and cell wall remodeling for successful colonization. Surprisingly, our data indicate that RALF‐mediated powdery mildew susceptibility is partially independent of FER and exerts its effect in a combination of signaling and structural functions. We propose that powdery mildew fungi require RALFs to provide a favorable growth environment, highlighting a new mechanism of host subversion by adapted plant pathogens.

## Materials and Methods

### Plant material and growth conditions

Plant lines used in this study are listed in Table S3. The genetic material used in this study is based on *Arabidopsis thaliana* (L.) *Heynh.*, accession Columbia‐0 (Col‐0). All plants were grown with two plants per pot in environmentally controlled conditions (21°C, 55–65% relative humidity, 8 h photoperiod). Five‐ to six‐week‐old plants were used for *Ecr* infection.

### Powdery mildew propagation

*Erysiphe cruciferarum* (*Ecr*) Opiz ex L. Junell 1967 was propagated weekly by transferring spores to 4–5‐wk‐old uninfected Col‐0 plants. Spores were used for further inoculation 3–4 wk after propagation. Propagation and experiments were performed in an environmentally controlled growth cabinet (22°C, 70% relative humidity, 12 h photoperiod).

### Molecular cloning

To generate p35S::RALF23 and p35::MLO2‐mCherry lines, gene sequences were cloned from gDNA using primers listed in Supporting Information Table S1 and assembled into a GoldenGate‐modified pCB302 binary vector for plant expression. The p35S::RALF23^Y93A/Y96A^ and p35S::RALF23^Y113A/Y114A^ constructs were obtained by site‐directed mutagenesis using primers listed in Table S1 and likewise assembled into a GoldenGate‐modified pCB302 binary vector. For pR23::mCherry‐RALF23 constructs, pR23 was cloned using primers listed in Table S1. Gene fragments for RALF23^WT^, RALF23^Y93A/Y96A^ and RALF23^Y113A/Y11A^ with an N‐terminal mCherry tag located between the signal peptide and the mature RALF23 were obtained by gene synthesis (Twist Bioscience, San Francisco, CA, USA). pR23 and the synthesized gene fragments were assembled into a GoldenGate‐modified pCB302 binary vector for plant expression. To generate CRISPR‐Cas9 mutants, the software tool chopchop (https://chopchop.cbu.uib.no/) was used to design target sites (one to two per gene of interest). All target sequences are listed in Table S1. Individual guide RNAs with gene‐specific target sites were obtained by gene synthesis (Twist Bioscience). Higher order gRNA stacks were cloned into pICSL4723OD with FastRed‐pRPS5::Cas9 (Castel *et al*., 2019). All generated plant expression constructs were transformed into *Agrobacterium tumefaciens* strain GV3101 before floral dip transformation.

### Conidiophore counting and fungal penetration assay

Conidiophore production was analyzed 5 d after *Ecr* infection. Leaves were destained using EtOH : Acetic Acid (6 : 1) and subsequently stained using ink (ink: 25% acetic acid, 9 : 1). Fungal structures were visualized using a Zeiss AXIO imager Z1.m microscope. Fungal penetration success was similarly analyzed and quantified at 2 days postinfection (dpi). A penetration attempt was scored as successful when the spore formed a penetration tube and first hyphal structures.

### Quantification of fungal DNA by qPCR

For quantification of fungal DNA, two infected plants per genotype were harvested at 5 dpi before DNA extraction with standard protocols. Reverse transcription quantitative polymerase chain reaction (RT‐qPCR) experiments were performed using Maxima SYBR green mix (Thermo Fisher Scientific, Waltham, MA, USA) on an AriaMx Real‐Time PCR system (Agilent Technologies, Santa Clara, CA, USA). The amount of fungal DNA was calculated relative to plant DNA using primers for the Arabidopsis small RuBisCO subunit (AtRbcS) and *Ecr* tubulin (Table S1).

### Re‐inoculation experiments

Five‐week‐old Col‐0, *fer‐4*, *llg1‐2* and CRISPR *ralf7x* plants were highly infected with powdery mildew. Eleven‐day‐old postinfection spores from these plants were used to infect 5‐wk‐old Col‐0.

### *Hyaloperonospora arabidopsidis* (*Hpa*) infection and quantification

For *Hpa* infection assays, plants were vernalized for 2–3 d in the dark at 4°C and grown in environmentally controlled conditions (22°C, 60% relative humidity, 10 h photoperiod). Fourteen‐day‐old seedlings were spray‐inoculated with a spore suspension of *Hpa* isolate Noco2 (5 × 10^4^ spores ml^−1^; Asai *et al*., 2015). After inoculation, plants were kept in trays covered with sealed lids to maintain high humidity (18°C, near‐saturation humidity). At 7 d postinoculation, infected aerial parts were harvested in water and vortexed to release spores. Spores were counted using a hemocytometer and normalized to leaf fresh weight.

### Infiltration experiments

Individual leaves were infiltrated with synthetic RALF23 (Sequence: ATRRYISYGALRRNTIPCSRRGASYYNCRRGAQANPYSRGCSAITRCRRS) 24 h before infection. Fusicoccin (10 μM), epigallocatechin gallate (EGCG) (50 μM) and MES buffers were infiltrated 2 h before *Ecr* infection.

### Gene expression analysis

For RNA extraction, TRIzol reagent (Roche) and purification with Direct‐zol™ RNA Miniprep Plus Kit (Zymo Research, Freiburg, Germany) with on‐column DNAse I digestion was used. RNA was reverse transcribed with random hexamer primer and Revert Aid reverse transcriptase (Thermo Fisher Scientific). RT‐qPCR experiments were performed using Maxima SYBR green mix (Thermo Fisher Scientific) on an AriaMx Real‐Time PCR system (Agilent Technologies). Gene expression levels were normalized to *UBIQUITIN5* (*UBQ5*). All primers are listed in Table S1.

### PME activity assay

PECTIN METHYL ESTERASE (PME) activity assays were performed as previously described (Bethke *et al*., 2014). In brief, plant material of untreated and infected plants was harvested 5 dpi and ground in liquid nitrogen. Plant powder was incubated with 500 μl protein extraction buffer (50 mM Tris–HCl pH 7.5, 150 mM NaCl, 10% glycerol, 5 mM dithiothreitol, 1% protease inhibitor cocktail, 2 mM Na_2_MoO_4_, 2.5 mM NaF, 1.5 mM activated Na_3_VO_4_, 1 mM phenylmethanesulfonyl fluoride and 0.5% IGEPAL) for 30 min at 8°C before centrifugation at 16 000 ***g*** for 30 min. The supernatant was transferred into a fresh 1,5‐ml tube, and protein concentration was determined by Bradford assay. The protein concentration was adjusted to 3 μg μl^−1^. To assess PME activity, 15 μl liquid was loaded into holes in an agarose gel plate containing esterified pectin (1.2% (w/v) agarose, 0.1% (w/v) pectin from apple, 12 mM citric acid and 50 mM Na_2_HPO_4_ pH 7) before incubation at 37°C for 16 h. Afterward, plates were briefly washed with water and stained with 0.05% ruthenium red for 30 min. Residual dye was washed off with water and images of the plates were captured by scanning. Darker stained areas correlating with PME activity were quantified using the Fiji ImageJ software (Schindelin *et al*., 2012).

### AHA‐phosphorylation analysis

Infected plant material was ground in liquid nitrogen. Proteins were extracted (3% [w/v] sodium dodecyl sulfate (SDS), 30 mM TRIS–HCl pH 8.0, 10 mM ethylenediaminetetraacetic acid (EDTA), 10 mM NaF, 30% [w/v] sucrose, 0.012% [w/v] Coomassie Brilliant Blue and 15% [v/v] 2‐Mercaptoethanol) and immediately used for SDS‐polyacrylamide gel electrophoresis (PAGE)/western blot. Proteins were detected using AHA/pThr947 antibodies (Hayashi *et al*., 2010). Band intensities were quantified using the Fiji ImageJ software.

### Quantification of salicylic acid, dihydroxybenzoic acids and camalexin

SA, DHBAs and camalexin were extracted as described (Nawrath & Métraux, 1999) with minor modifications. Ground fine leaf powder was spiked with 250 ng *ortho*‐anisic acid (oANI) as internal standard and sequentially extracted with 70% and 90% methanol for 1 h at 65°C. Combined supernatants were concentrated, macromolecules precipitated with 5% trichloroacetic acid, and supernatants partitioned two times against cyclohexane/ethyl acetate (1 : 1) to obtain free SA, DHBAs, and camalexin. The aqueous phase was acidified with 8 M HCl (1 : 1) and incubated for 1 h at 80°C. Hydrolyzed extracts were spiked with oANI and partitioned as above to obtain glycosylated SA and DHBAs. Organic phases of free and glycosylated fractions were concentrated and dissolved in 90% 25 mM KH_2_PO_4_, pH 2.6/10% acetonitrile (ACN). HPLC analysis was performed on an Agilent 1260 Infinity II system with fluorescence detection. SA, DHBAs, camalexin and oANI were separated on an Agilent InfinityLab Poroshell 120 SB‐C18 column (3 × 100 mm, 2.7 μm) at 40°C, using a solvent gradient of (A) 25 mM KH_2_PO_4_, pH 2.6 and (B) ACN:H_2_O (99 : 1) at 1 ml min^−1^ flow rate (all steps in v/v): 0 to 1 min: 10% B; 1 to 6 min: 10–25% B; 6 to 8.5 min: 25–80% B; 8.5 to 10.5 min: 80% B; 10.5 to 11 min: 80–10% B; 11 to 14 min: 10% B. SA (Ex 305 nm, Em 407 nm), DHBAs (Ex 320 nm, Em 450 nm), camalexin (Ex 318 nm, Em 370 nm) and oANI (Ex 305 nm, Em 365 nm) were calibrated against a standard dilution series of all metabolites of interest in the range of 1–100 ng.

### Histochemical staining of callose deposition in rosette leaves and trichomes

Three rosette leaves per 35‐d‐old Arabidopsis plant were collected and destained in 70% ethanol (v/v). Afterward, leaves were incubated overnight in aniline blue solution (0.01% Aniline Blue (m/v) in 150 mM K_2_HPO_4_ buffer) and mounted on a microscopy slide. Micrographs were captured by a BZ‐9000 microscope (Keyence, Osaka, Japan) using UV illumination. Images were optimized by Adobe Photoshop 2024 in an identical manner for all micrographs. Trichome and trichome branch number were scored by counting.

### Callose quantification at the fungal penetration site

Leaves were harvested and destained in EtOH:Acetic Acid (6 : 1). Next, leaves were incubated for 10 min in 0.067 mM K_2_HPO_4_. Subsequently, leaves were transferred into staining solution with 0.05% methyl blue in 0.067 M K_2_HPO_4_and incubated overnight. Fungal structures were stained before analysis with 0.05% propidium iodide (PI). Methyl blue fluorescence was detected between 480 and 530 nm with a laser at 458 nm for excitation. PI fluorescence was excited with a 510‐nm laser and detected at 600–620 nm. Z‐Projections were created, and mean gray values were measured using Fiji (Schindelin *et al*., 2012).

### COS‐488 synthesis, imaging and quantification

COS‐488 was synthesized as described before (Mravec *et al*., 2017). Chitosan oligosaccharides (Carbosynth OC09272) were dissolved in 100 mM NaCH_3_CO_2_ (pH 4.9) to 1 mg/mL. AlexFluor 488 hydroxylamine was prepared in dimethyl sulfoxide (10 mg/mL). A total of 0.16 μl were added to 500 μl COS solution and incubated at 37°C in dark for 2 d under gentle shaking. Staining of leaf epidermis cells was performed by harvesting leaf disks from mature Arabidopsis plants. COS‐488 was infiltrated at 1 : 1000 dilution in ½‐strength Murashige and Skoog medium (½ MS) + 1% Sucrose pH 5.8 under mild vacuum. Afterward, leaf disks were incubated in COS‐488 solution for 2 h and washed with ½ MS + 1% sucrose pH 5.8 three times under mild vacuum. Samples were mounted and z‐stacks acquired using a Leica SP8 confocal microscope equipped with HC PL APO 40x water objective, argon laser and HyD detectors. COS‐488 was excited at 488 nm. Emission was detected between 490 and 560 nm. Z‐Projections were created, and mean gray values were measured using Fiji (Schindelin *et al*., 2012).

### Confocal microscopy

Confocal laser‐scanning microscopy was performed using a Leica TCS SP5 (Leica, Germany) microscope (with Leica Application Suite X 3.7.4.23463). All images were taken with a 20× water immersion objective. To visualize green fluorescent protein (GFP) fluorescence, an argon laser with HyD detector was used (emission at 488 nm, detection 500–550 nm). mCherry fluorophore was excited with a DPSS laser (emission 561 nm, detection 600–640 nm). Changes in the apoplastic pH around fungal infection sites were analyzed using pUBQ10::SYP122‐pHusion expressing plants (Kesten *et al*., 2019). GFP and red fluorescent protein (RFP) were excited with a 488‐nm and a 561‐nm laser, respectively. Signals were detected at 500–545 nm (GFP) and 650–695 nm (RFP). Z‐stacks with identical settings were taken at the penetration site and in unpenetrated areas. The GFP/RFP ratios were determined using Fiji ImageJ.

### Co‐IP‐MS

Sample preparation and mass spectrometric data acquisition for all co‐immunoprecipitation‐mass spectrometry (Co‐IP) samples was carried out as previously described (Scheinost *et al*., 2026). Protein identification and quantification were performed with the MaxQuant software, using parameters previously described (Scheinost *et al*., 2026) and the following exceptions: Proteins were identified by searching MS2 spectra against the TAIR protein database for *Arabidopsis thaliana* (downloaded March 2022, 48 231 protein entries). Phosphorylation of serine/threonine/tyrosine, oxidation of methionine and acetylation at the protein N‐terminus were specified as variable modifications. The ‘match‐between‐run’ functionality was enabled (matching time window 0.7 min, alignment time window 20 min). For protein quantification, the MaxQuant output table proteinGroups.txt and Label‐Free Quantification (LFQ; Cox *et al*., 2014) intensities were used. Only proteins detected in at least two biological replicates in at least one experimental condition were further processed. Missing values were imputed by a protein‐specific constant value, which was defined as the lowest detected protein‐specific LFQ‐value over all samples divided by two, using the data visualization platform omicsViewer (DOI: 10.1101/2022.03.10.483845). Additionally, a maximal imputed LFQ value was defined as the 15% quantile of the protein distribution from the dataset. The volcano plots (Figs S6A, S7A) were generated using RStudio. Interactions were marked as significant if they passed the threshold of FDR < 0.05 and fold change > 2. The FER‐GFP infected with *Ecr* and untreated experiments were performed in biological triplicates, while all other experiments were performed in biological quadruplets.

### Identification of putative powdery mildew RALF‐likes

To identify putative RALF‐like peptides in the predicted proteins and genome assemblies of selected phytopathogenic fungi, we used the hmmsearch program from the hmmer suite v.3.3.2 (www.hmmer.org) with default parameters. The hidden markov model profile (.hmm) for the RALF motif (PF05498.15) was obtained from the PFAM website (https://ftp.ebi.ac.uk/pub/databases/Pfam/current_release/Pfam‐A.hmm.gz), access data 19 November 2023. RALF HMM profile was used to search predicted protein sequences from the selected fungal species (see Table S4) using the hmmsearch function. Hits with E‐values below 1e^−05^ were retained. To perform the hmmsearch analysis on the genome assemblies, we used a custom Python script to split the contigs into 300‐bp fragments with a sliding window of 50 bp. All six open reading frames of these split sequences were translated and stop codons were removed to generate a multi‐FASTA file suitable for be processed with hmmsearch. Search hits with *E*‐values <1e^−05^ were retained. Accession numbers of genome assemblies used in this analysis are reported in Table S4.

### Quantification and statistical analysis

GraphPad Prism (v.8.0.1) was used to perform statistical analysis. Detailed descriptions of the sample size, *P*‐values and statistical methods are indicated in the respective figure legends.

## Results

### FER, LLGs and LRX are powdery mildew susceptibility factors

**Fig. 1:**
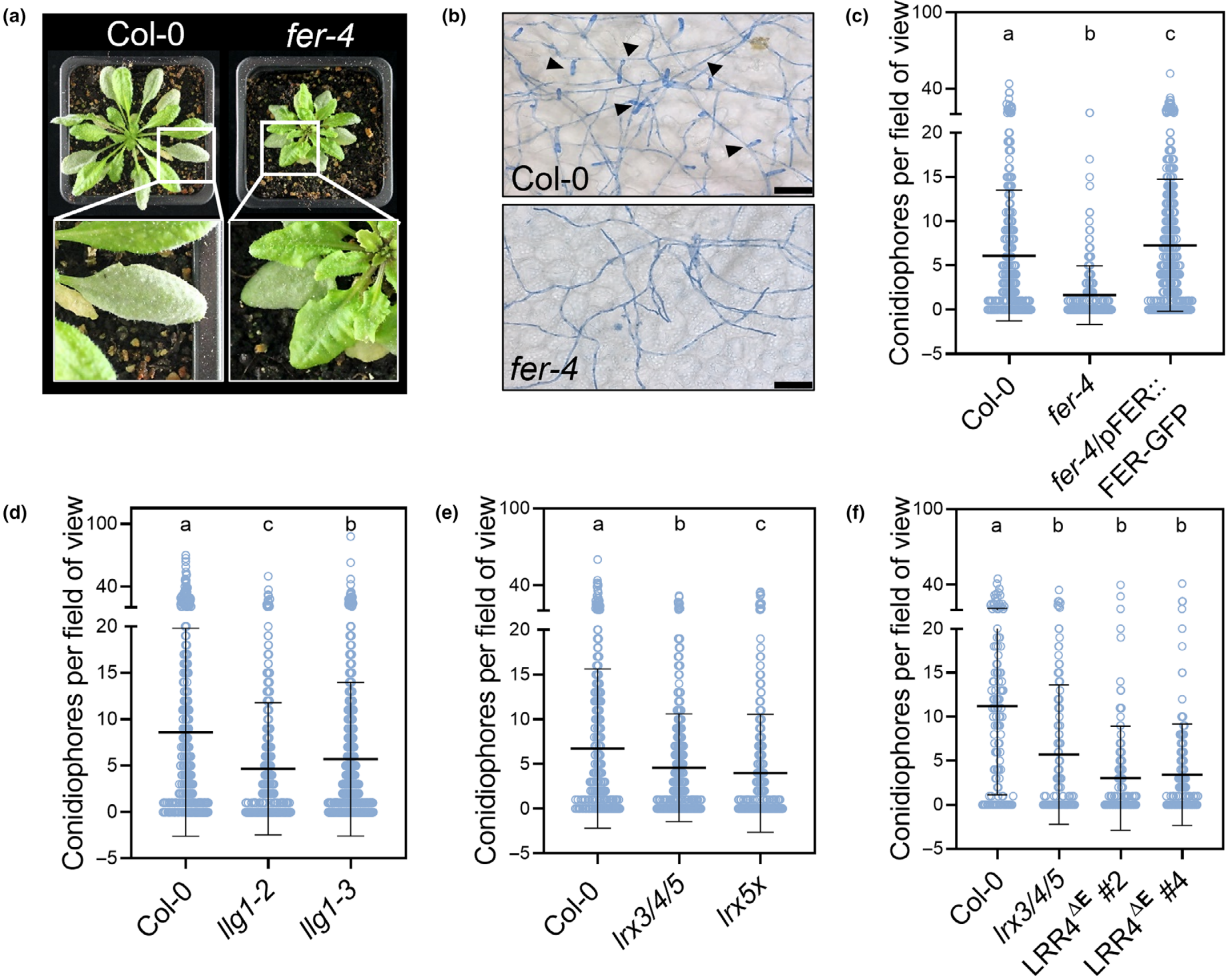
FER and additional RALF‐binding proteins are powdery mildew susceptibility factors in Arabidopsis. (a) Images of Columbia‐0 (Col‐0) and *fer‐4* plants infected with *Ecr* (14 dpi). (b) Ink stained *Ecr* colonies (5 dpi) grown on Col‐0 and *fer‐4* plants. Black arrows indicate conidiophores. Bars, 50 μm. (c) Conidiophores per field of view (5 dpi) of fungal colonies grown upon *Ecr* infection of the indicated genotypes. Mean ± SD, *n* = 223–392 pooled from three independent experiments (Dunn's multiple comparisons test, a‐b, b‐c *P* < 0.0001; a‐c *P* = 0.0106). (d) Conidiophores per field of view (5 dpi) of fungal colonies grown upon *Ecr* infection of the indicated genotypes. Mean ± SD, *n* = 345–665 pooled from five independent experiments (Dunn's multiple comparisons test, a‐b, a‐c *P* < 0.0001; b‐c *P* = 0.0235). (e) Conidiophores per field of view (5 dpi) of fungal colonies grown upon *Ecr* infection of the indicated genotypes. Mean ± SD, *n* = 285–453 pooled from three independent experiments (Dunn's multiple comparisons test, a‐b *P* = 0.0150; a‐c *P* < 0.0001; b‐c *P* = 0.0408). (f) Conidiophores per field of view (5 dpi) of fungal colonies grown upon *Ecr* infection of the indicated genotypes (LRR4^ΔE^: LRX4 lacking the extensin domain). Mean ± SD, *n* = 113–159 pooled from two independent experiments (Dunn's multiple comparisons test, a‐b *P* < 0.0001). All experiments were done at least three times with similar results, except F, which was repeated once with identical results. Dpi, days post infection; *Ecr*, *Erysiphe cruciferarum*; FER, FERONIA; LLG, LORELEI‐LIKE GPI‐ANCHORED PROTEIN; LRX, LEUCINE‐RICH REPEAT EXTENSIN; LRR, LEUCINE‐RICH REPEAT; GFP, GREEN FLUORESCENCE PROTEIN; RALF, RAPID ALKALINIZATION FACTOR.

Ler‐0 *fer‐1* is more resistant to *Golovinomyces orontii* (Kessler *et al*., 2010). We confirmed this phenotype in Arabidopsis Col‐0 using the *fer‐4* mutant allele (Duan *et al*., 2010) and *Ecr* (Micali *et al*., 2008). The *fer‐4* mutant showed slightly weaker visible symptoms (Fig. 1a), reduced conidiophore production and less relative fungal DNA content compared with Col‐0 5 dpi (Fig. 1b,c, S1A). Expression of pFER::FER‐GFP complemented the *fer‐4* resistance phenotype (Fig. 1c).

Next, we investigated whether FER‐related RALF receptor complex components are involved in *Ecr* susceptibility. The *llg1‐2* mutant showed reduced conidiophore production (Fig. 1d). A similar effect was observed in the *llg1‐3* allele, in which LLG1 is impaired in RALF binding, but mutants do not show growth phenotypes (Figs 1d, S2; Shen *et al*., 2017; Xiao *et al*., 2019). The resistance phenotype of the *llg1* mutants, however, was not clear in qPCR quantification (Fig. S1B), indicating potential redundancy of LLG1 with related members of the LRE/LLG gene family (Xiao *et al*., 2019; Noble *et al*., 2022). Surprisingly, *llg1‐2* and *llg1‐3* were previously described as more susceptible to *Golonivomyces cichoracearum* (Shen *et al*., 2017), raising the question of mildew‐species‐dependent differences in *llg1* pathophenotypes, unlike *fer* (Fig. 1c,d; Kessler *et al*., 2010). RALFs also bind to cell wall localized LRX proteins (Moussu *et al*., 2020). A *lrx3 lrx4 lrx5* (*lrx3/4/5*) triple mutant phenocopies *fer* in immune signaling, salt stress responses and plant morphology (Zhao *et al*., 2018; Dünser *et al*., 2019; Moussu *et al*., 2020; Gronnier *et al*., 2022). This *lrx3/4/5* mutant displayed reduced conidiophore production upon *Ecr* infection (Fig. 1e). Yet, relative fungal DNA content was unaltered (Fig. S1C). Further mutating *LRX1* and *LRX2* in *lrx3/4/5* (*lrx1/2/3/4/5*, hereafter *lrx5x*; Herger *et al*., 2020) promoted the resistance phenotype (Figs 1e, S1C). Overexpression of LRR4‐citrine (LRR4^ΔE^, LRX4 lacking the extensin domain) has a dominant negative effect on plant growth, while PTI responses are not affected (Dünser *et al*., 2019; Gronnier *et al*., 2022). The p35S::LRR4‐citrine lines phenocopied *lrx5x* and *fer*‐*4* in showing reduced *Ecr* conidiophore production (Fig. 1f).

### Powdery mildew requires endogenous RALF peptides for successful colonization

**Fig. 2:**
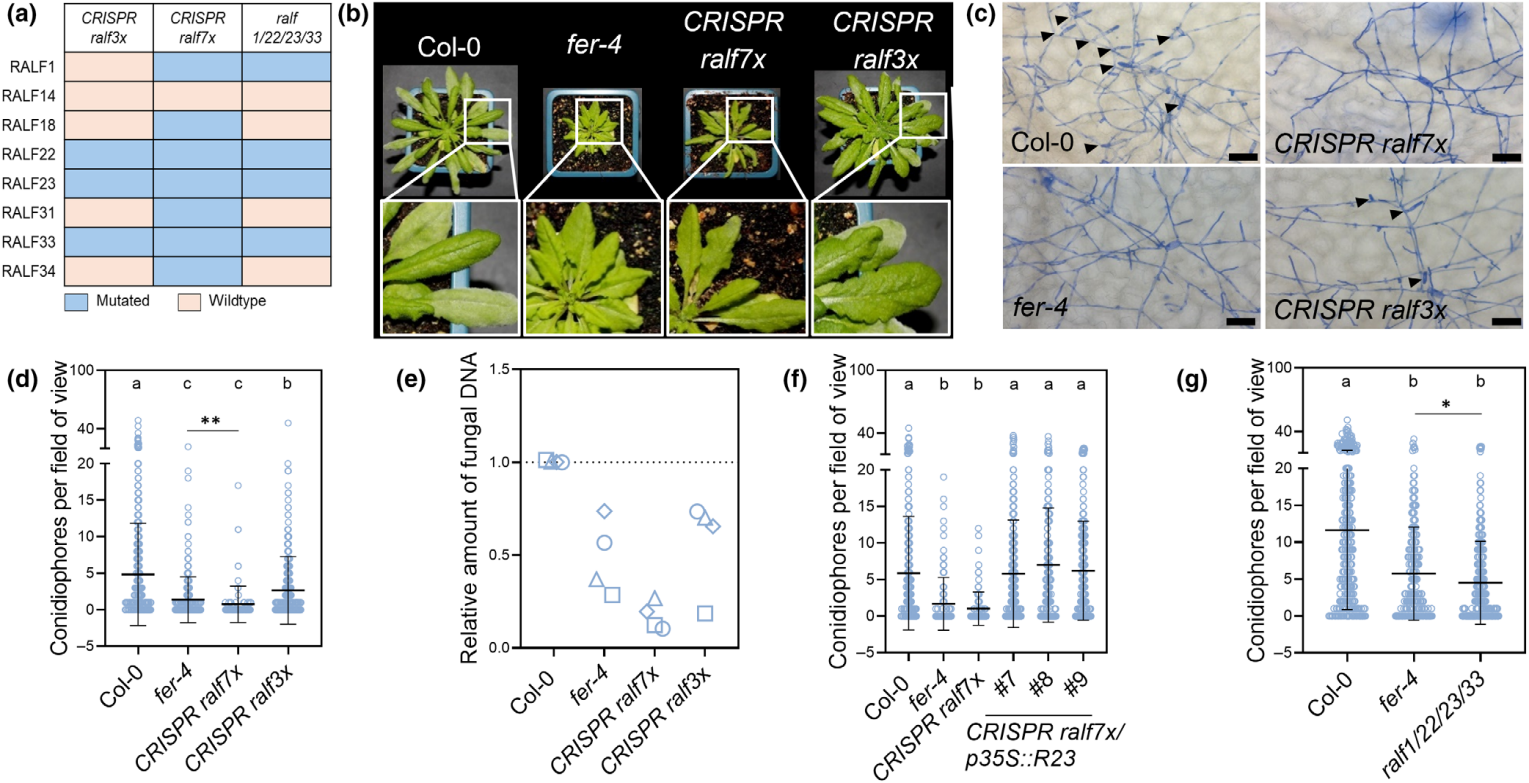
Specific RALFs are powdery mildew susceptibility factors in Arabidopsis. (a) Table of RALF peptides mutated in CRISPR *ralf3x*, CRISPR *ralf7x* and *ralf1/22/23/33*. (b) Images of Columbia‐0 (Col‐0), *fer‐4*, CRISPR *ralf3x* and CRISPR *ralf7x* plants infected with *Ecr* (14 dpi). (c) Ink stained *Ecr* colonies (5 dpi) grown on Col‐0, *fer*‐*4*, CRISPR *ralf3x* and CRISPR *ralf7x* plants. Black arrows indicate conidiophores. Bars, 50 μm. (d) Conidiophores per field of view (5 dpi) of fungal colonies grown upon *Ecr* infection of the indicated genotypes. Mean ± SD, *n* = 98–456 pooled from three independent experiments (Dunn's multiple comparisons test, a‐b, a‐c *P* < 0.0001, b‐c *P* < 0.0002. Comparison between *fer‐4* and CRISPR *ralf7x*: Mann–Whitney test, **, *P* = 0.007). (e) Amount of fungal DNA normalized to plant DNA (5 dpi) upon *Ecr* infection of the indicated genotypes, *n* = 4, data points with different symbols indicate independent biological replicates. (f) Conidiophores per field of view (5 dpi) of fungal colonies grown upon *Ecr* infection of the indicated genotypes. Mean ± SD, *n* = 101–286 pooled from three independent experiments (Dunn's multiple comparisons test, a‐b *P* < 0.0001). (g) Conidiophores per field of view (5 dpi) of fungal colonies grown upon *Ecr* infection of the indicated genotypes. Mean ± SD, *n* = 226–382 pooled from three independent experiments (Dunn's multiple comparisons test, a‐b *P* < 0.0001. Comparison between *fer‐4* and *ralf1/22/23/33*: Mann–Whitney test, **, *P* = 0.0132). All experiments were performed at least three times with similar results. Dpi, days postinfection; *Ecr*, *Erysiphe cruciferarum*; FER, FERONIA; RALF, RAPID ALKALINIZATION FACTOR.

We hypothesized that RALF signaling through FER‐LLG1 and/or their binding to LRXs is required for *Ecr* colonization. Many plant pathogens secrete RALF peptide mimics to promote virulence (Masachis *et al*., 2016; Thynne *et al*., 2017; Zhang *et al*., 2020). We searched available genomes of powdery mildew species for the presence of potential RALFs. However, consistent with previous reports (Thynne *et al*., 2017), we could not detect evidence for RALFs among different *Erysiphales* species (Table S4, see the Materials and Methods section for details on our bioinformatic search pipeline). Thus, we hypothesized that endogenous RALFs are involved in the *Ecr‐*Arabidopsis interaction. Arabidopsis encodes for 37 RALFs (Abarca *et al*., 2021). We anticipated genetic redundancy and generated CRISPR mutants in which we targeted *RALF23*, a described FER‐LLG1 ligand (Stegmann *et al*., 2017; Xiao *et al*., 2019; J. Tang *et al*., 2022), and seven additional RALF peptides with predicted leaf expression (*RALF1*, *RALF14*, *RALF18*, *RALF22*, *RALF23*, *RALF31*, *RALF33* and *RALF34*; Fig. S3A). We could not obtain an octuple mutant but generated two higher order mutants, *ralf22/23/33* (CRISPR *ralf3x*) and *ralf1/18/22/23/31/33/34* (CRISPR *ralf7x*; Figs 2a, S3B,C). CRISPR *ralf3x* displayed a mild growth phenotype with slightly shortened petioles, while CRISPR *ralf7x* was strongly impaired in shoot growth (Figs 2b, S2, S3D). Compared with *fer‐4*, however, CRISPR *ralf7x* showed less pronounced shoot growth defects with a trend toward longer petioles (Fig. S3D) and an overall more wild‐type‐like morphology (Figs 2b, S2). *Ecr* infection was reduced on both CRISPR *ralf3x* and CRISPR *ralf7x* (Fig. 2b–e). Notably, CRISPR *ralf7x* displayed slightly elevated resistance compared with *fer‐4* (Fig. 2d). Expression of *p35S::RALF23* complemented CRISPR *ralf7x* growth and *Ecr* infection phenotypes (Figs 2f, S2, S3E,F). Additionally, the previously reported *ralf1/22/23/33* quadruple mutant (Lan *et al*., 2023) was more resistant to *Ecr* with slightly less condiophore production compared with *fer‐4* (Fig. 2a,g). We analyzed expression of the seven *RALFs* mutated in CRISPR *ralf7x* in Col‐0 upon *Ecr* infection by qPCR. *RALF18* and *RALF23* were significantly upregulated at 4 and 5 dpi (Fig. S4). Together with our genetic data, this underlines the role of endogenous RALF peptides in *Ecr* host colonization.

### FER and RALF mutants show postpenetration powdery mildew resistance

We next sought out to unravel cellular mechanisms underlying RALF‐mediated *Ecr* susceptibility. RALF‐induced calcium influx in synergid cells and pollen tubes is facilitated by FER‐LRE‐mediated activation of the NTA/MLO7 calcium channel (Gao *et al*., 2022). The MLO family encodes important powdery mildew susceptibility factors across families (Büschges *et al*., 1997; van Schie & Takken, 2014; Acevedo‐Garcia *et al*., 2017; Nekrasov *et al*., 2017). In Arabidopsis, mutation of three MLOs is required to confer full penetration resistance (Consonni *et al*., 2006).

MLO‐YFP in barley and MLO2‐GFP in Arabidopsis focally accumulate at powdery mildew penetration sites (Bhat *et al*., 2005; Huebbers *et al*., 2024). We confirmed MLO2 localization to the fungal penetration site with p35S::MLO2‐mCherry lines (Fig. S5A, left panel). We tested whether FER‐GFP accumulates at *Ecr* penetration sites using *fer‐4 pFER::FER‐GFP* (Duan *et al*., 2010). Although FER‐GFP signal outlined the plasma membrane, specific penetration site recruitment was not detected (Fig. S5A, right panel). The *fer‐4* mutant showed strongly reduced production of conidiophores on infected tissue (Fig. 1c), but initial hyphal growth was largely unaffected (Fig. S5B). Also, epidermal cell penetration was unaffected in *fer‐4* and CRISPR *ralf7x*, unlike *mlo2/6/12* (Fig. S5C). Callose deposition was not enhanced at the penetration site on *fer‐4* or CRISPR *ralf7x* compared with Col‐0 (Fig. S5D,E). This suggests that mutating RALF‐related components primarily affects *Ecr* reproductive success and not fungal entry or epiphytic growth. We also tested whether *Ecr* spores derived from colonies grown on *fer‐4*, CRISPR *ralf7x* and *llg1‐2* mutants show compromised infection potential upon re‐inoculation of a Col‐0 host. However, neither of the spores displayed altered pathogenicity compared with Col‐0‐derived spores (Fig. S5F). This indicates that, although colonies grown on the mutant plants have a reduced number of spores, their quality is not impaired.

The *mlo2/6/12* mutant displays aberrant callose deposition in trichomes with delocalized or absent accumulation (Huebbers *et al*., 2024). Likewise, *fer‐4* mutants are impaired in trichome formation (Duan *et al*., 2010). Consistently, we observed fewer trichomes on *fer‐4* but not on CRISPR *ralf7x* (Fig. S5G). Unlike *mlo2/6/12*, neither CRISPR *ralf7x* nor *fer‐4* showed misregulated trichome callose deposition (Fig. S5H; Huebbers *et al*., 2024). A fraction of trichomes on the CRISPR *ralf7x* mutant were enlarged in the base region, which was not observed on Col‐0 and *fer‐4* (Fig. S5H). Furthermore, trichomes on *fer‐4* and CRISPR *ralf7x* had fewer and more branches, respectively, compared with Col‐0 (Fig. S5I). Collectively, these data suggest that FER‐RALF‐dependent *Ecr* susceptibility and trichome development is most likely not directly linked to MLO function.

### The powdery mildew resistance phenotype of FER pathway mutants is not associated with deregulated defense phytohormone signaling

**Fig. 3:**
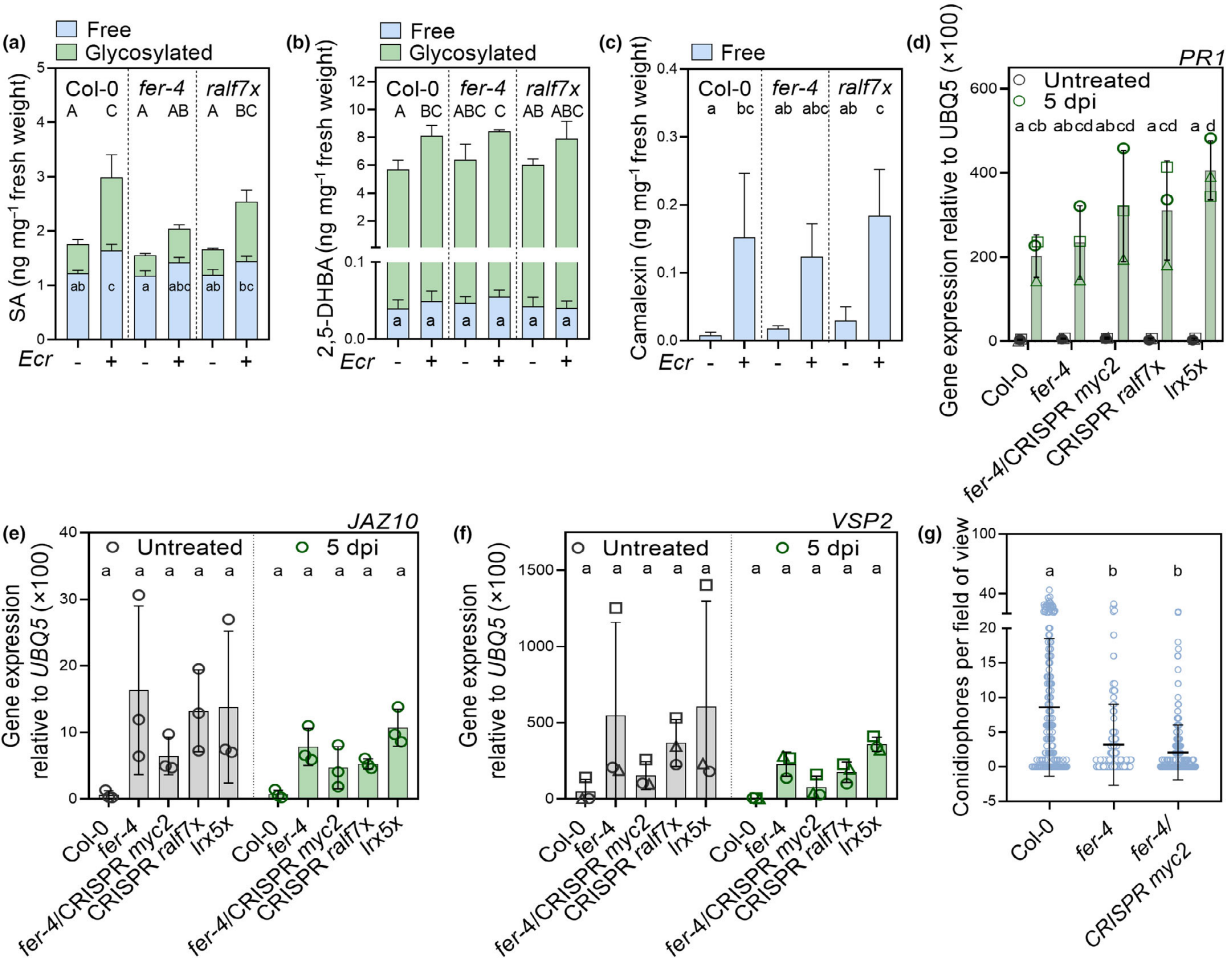
FER‐RALF‐mediated powdery mildew susceptibility in Arabidopsis is independent of JA and SA signaling. (a) Quantification of free (blue) and glycosylated (green) SA in untreated and *Ecr* infected (5 dpi) leaves of the indicated genotypes. Mean ± SD, *n* = 3 pooled from three independent experiments (Tukey's multiple comparisons test, free and glycosylated SA was analyzed individually, free: a‐bc *P* = 0.0423; a‐c *P* = 0.0006; ab‐c *P* ≤ 0.0013, glycosylated: A–C *P* ≤ 0.0029; A–BC *P* ≤ 0.0383). (b) Quantification of free (blue) and glycosylated (green) 2,5‐DHBA in untreated and *Ecr* infected (5 dpi) leaves of the indicated genotypes. Mean ± SD, *n* = 3 pooled from three independent experiments (Tukey's multiple comparisons test, free and glycosylated 2,5‐DHBA was analyzed individually, free: not significant, glycosylated: A‐C *P* = 0.0153; A‐BC *P* = 0.0342; AB‐C *P* = 0.0345). (c) Quantification of free (blue) Camalexin in untreated and *Ecr* infected (5 dpi) leaves of the indicated genotypes. Mean ± SD, *n* = 3 pooled from three independent experiments (Tukey's multiple comparisons test, a‐bc *P* = 0.0486; ab‐c *P* ≤ 0.0319; a‐c *P* = 0.0137. (d) Reverse transcription quantitative polymerase chain reaction (RT‐qPCR) of *PR1* in untreated (gray) adult leaves and upon infection with *Ecr* (5 dpi, green). Housekeeping gene *UBQ5*. Mean ± SD, *n* = 3, data points with different symbols indicate independent biological replicates (Tukey's multiple comparisons test, a‐d *P* < 0.0001; a‐cb *P* ≤ 0.0464; a‐cd *P* ≤ 0.0137; ab‐cd *P* ≤ 0.0161). (e) RT‐qPCR of *JAZ10* in untreated (gray) adult leaves and upon infection with *Ecr* (5 dpi, green). Housekeeping gene *UBQ5*. Mean ± SD, *n* = 3, data points with different symbols indicate independent biological replicates (Tukey's multiple comparisons test, not significant) (f) RT‐qPCR of *VSP2* in untreated (gray) adult leaves and upon infection with *Ecr* (5 dpi, green). Housekeeping gene *UBQ5*. Mean ± SD, *n* = 3, data points with different symbols indicate independent biological replicates (Tukey's multiple comparisons test, not significant). (g) Conidiophores per field of view (5 dpi) of fungal colonies grown upon *Ecr* infection of the indicated genotypes. Mean ± SD, *n* = 152–209 pooled from three independent experiments (Dunn's multiple comparisons test, a‐b *P* < 0.0001). All experiments were performed three times with similar results. Dpi, days post infection; *Ecr*, *Erysiphe cruciferarum*; FER, FERONIA; LRX, LEUCINE‐RICH REPEAT EXTENSIN; RALF, RAPID ALKALINIZATION FACTOR; SA, salicylic acid; 2,5‐DHBA, dihydroxybenzoic acid; UBQ5, UBIQUITIN 5; PR1, PATHOGENESIS‐RELATED 1; JAZ10, JASMONATE‐ZIM‐DOMAIN PROTEIN; VSP2, VEGETATIVE STORAGE PROTEIN.

Salicylic acid (SA) is an important defense hormone promoting resistance to biotrophic and hemibiotrophic pathogens (Peng *et al*., 2021). Ler‐0 *fer‐1* seedlings did not show elevated basal *PATHOGENESIS‐RELATED 1* (*PR1*) expression, an SA marker gene (Kessler *et al*., 2010). Yet, the weak *fer‐5* allele in Col‐0 showed mildly constitutively elevated SA levels in seedlings (Duan *et al*., 2010; Engelsdorf *et al*., 2018), prompting us to test whether *fer‐4's Ecr* resistance phenotype may be associated with elevated SA levels. We quantified free and glycosylated SA by high‐performance liquid chromatography (HPLC) upon *Ecr* infection. *Ecr* infection led to mildly enhanced SA levels (Fig. 3a). However, neither *fer‐4* nor CRISPR *ralf7x* showed higher SA levels before or after infection (Fig. 3a). We also measured the major catabolic SA products 2,3‐DHBA and 2,5‐DHBA to investigate the flux through the SA pathway (Zhang *et al*., 2013, 2017) as well as camalexin levels associated with antifungal resistance (Liu *et al*., 2016; Mucha *et al*., 2019). Neither of those compounds were enhanced before or after infection in *fer‐4* and CRISPR *ralf7x* (Figs 3b,c, S6A). *PR1* expression was also not elevated in any of the mutants in control conditions. *Ecr* infection resulted in enhanced *PR1* expression. The *lrx5x* mutant showed enhanced *Ecr*‐induced *PR1* expression, which, however, was not observed in the other mutants (Fig. 3d). When infected with the biotrophic oomycete pathogen *Hpa, fer‐*4 and CRISPR *ralf7x* were more susceptible than Col‐0 or *fer*‐4/*pFER::FER‐GFP* (Fig. S6B). This further supports the hypothesis that reduced *Ecr* sporulation observed on the tested mutants is not caused by enhanced SA signaling.

Jasmonic acid (JA) is usually implicated in defense against insect herbivory and necrotrophic fungi (Bürger & Chory, 2019), but JA treatment can also inhibit colonization by the powdery mildew fungus *Erysiphe cichoracearum* (Zimmerli *et al*., 2004). FER inhibits JA signaling by destabilizing the transcription factor MYC2 (Guo *et al*., 2018). We generated a CRISPR‐Cas9 *myc2* mutant in *fer‐4* that abolished *MYC2* expression (Fig. S6C,D). Similar to previous reports, *fer‐4* CRISPR *myc2* partially restored the *fer‐4* growth phenotype (Fig. S6E; Guo *et al*., 2018). In *fer‐4*, the CRISPR *myc2* mutation showed a clear trend toward complementation of deregulated JA‐responsive gene expression (Guo *et al*., 2018), a phenotype that was also observed in CRISPR *ralf7x* and *lrx5x* (Figs 3e,f, S6D). Importantly, mutating *MYC2* did not restore *Ecr* susceptibility in *fer‐4* (Fig 3g, S6F). This suggests that the resistance phenotype of *fer‐4* is independent of MYC2‐mediated JA signaling.

### Apoplastic pH modulations affect powdery mildew infection success

To determine potential protein interaction partners of FER, we performed Co‐IP‐MS in mature leaves of *fer‐4* pFER::FER‐GFP lines. We identified 68 potential interaction partners of FER‐GFP, with the majority being RLKs or transport‐related proteins (Fig. S7A,B). We identified four CrRLK1L members (HERK1, MDS1/LET2, THE1 and LET1). In response to *Ecr* infection, we could only detect six differential FER‐GFP interactors, which may be related to the early experimental time point chosen (1 dpi; Fig. S8A,B).

**Fig. 4:**
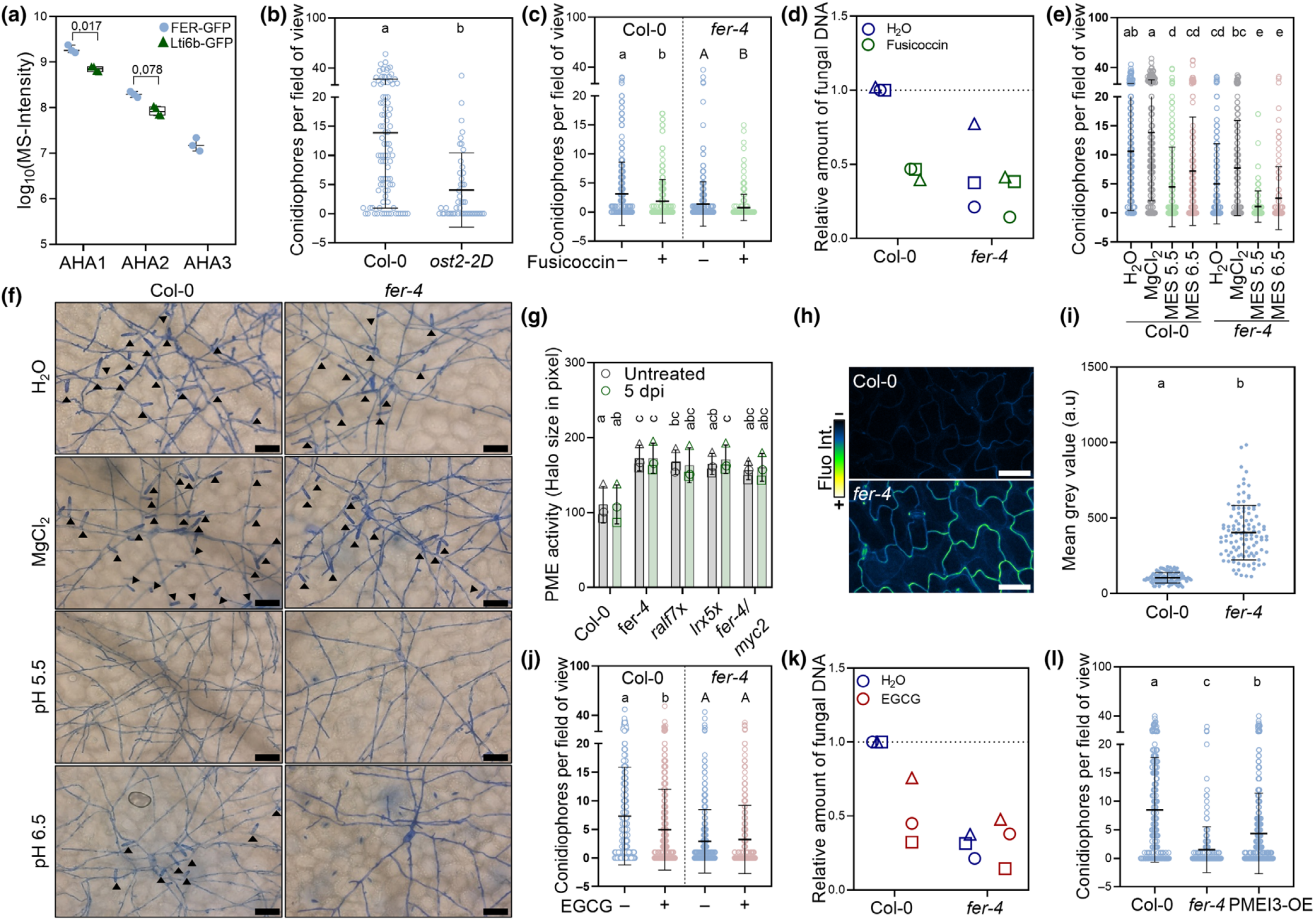
Apoplastic pH modulation and PME activity determine powdery mildew infection success on Arabidopsis. (a) Mass spectrometry (MS) intensities measured after co‐immunoprecipitation with untreated pFER::FER‐GFP and p35S::Lti6b‐GFP plants using GFP‐TRAP beads. *n* = 3 for pFER::FER‐GFP, *n* = 4 for p35S::Lti6b‐GFP, data points indicate independent biological replicates. FDR values indicate statistical differences between pFER::FER‐GFP and p35S::Lti6b‐GFP. No AHA3 derived peptides were detected in p35S::Lti6b‐GFP samples. (b) Conidiophores per field of view (5 dpi) of fungal colonies grown upon *Ecr* infection of the indicated genotypes. Mean ± SD, *n* = 49–91 pooled from two independent experiments (Mann–Whitney test, a‐b *P* < 0.0001). (c) Conidiophores per field of view (5 dpi) of fungal colonies grown upon *Ecr* infection of leaves pretreated with H_2_O (blue) or Fusicoccin (green). Mean ± SD, *n* = 232–316 pooled from seven independent experiments (Mann–Whitney test, genotypes were analyzed separately, Col‐0: a‐b *P* = 0.0058, *fer‐4*: A‐B *P* = 0.002). (d) Amount of fungal DNA normalized to plant DNA (5 dpi) upon *Ecr* infection and pretreatment with H_2_O (blue) or Fusicoccin (green). *n* = 3, data points with different symbols indicate independent biological replicates. All data are normalized to Col‐0 (H_2_O). (e) Conidiophores per field of view (5 dpi) after pretreatment with H_2_O (blue), MgCl_2_ 15 mM pH 7 (gray), MES buffer 10 mM pH 5.5 (green) and MES buffer 10 mM pH 6.5 (red) before *Ecr* infection. Mean ± SD, *n* = 126–195 pooled from three independent experiments (Dunn's multiple comparison, a‐bc, a‐dc, a‐d, a‐e, ab‐d, ab‐e, bc‐3 *P* < 0.0001, ab‐cd *P* ≤ 0.0086, bc‐d *P* = 0.0049, d‐e *P* ≤ 0.034). (f) Ink stained *Ecr* colonies (5 dpi) grown on Col‐0 and *fer‐4* plants after pretreatment with 15 mM MgCl_2_, 10 mM MES buffer (pH 5.5) and 10 mM MES buffer (pH 6.5). Black arrows indicate conidiophores. Bars, 50 μm. (g) PME activity as ruthenium red stained halo size in pixel in untreated leaves (gray) and upon *Ecr* infection (5 dpi, green) of the indicated genotypes. Mean ± SD, *n* = 3, data points with different symbols indicate independent biological replicates (Tukey's multiple comparisons test, a‐c *P* ≤ 0.0292, a‐bc *P* = 0.0484, ab‐c *P* ≤ 0.0312). (h) Representative confocal images of epidermal cells of Col‐0 and *fer‐4* leaves stained with COS‐488 labeling de‐esterified pectin. Bars, 50 μm. (i) Quantification of COS‐488 fluorescence intensity in Col‐0 and *fer‐4* leaves. Mean ± SD, *n* = 100–110 pooled from two independent experiments (Unpaired *t*‐test, a‐b *P* < 0.0001). (j) Conidiophores per field of view (5 dpi) of fungal colonies grown upon *Ecr* infection of leaves pretreated with H_2_O (blue) or EGCG (red). Mean ± SD, *n* = 235–376 pooled from seven independent experiments (Mann–Whitney test, genotypes were analyzed separately, Col‐0: a‐b *P* < 0.0001, *fer‐4*: ns). (k) Amount of fungal DNA normalized to plant DNA (5 dpi) upon *Ecr* infection and pretreatment with H_2_O (blue) or EGCG (red). *n* = 3, data points with different symbols indicate independent biological replicates. All data are normalized to Col‐0 (H_2_O). (l) Conidiophores per field of view (5 dpi) of fungal colonies grown upon *Ecr* infection of the indicated genotypes. Mean ± SD, *n* = 182–309 pooled from four independent experiments (Dunn's multiple comparisons test, a‐b, b‐c, a‐c *P* < 0.0001). All experiments were performed at least three times with similar results. AHA, AUTOINHIBITED H^+^‐ATPase; Dpi, days post infection; *Ecr*, *Erysiphe cruciferarum*; FER, FERONIA; GFP, GREEN FLUORESCENCE PROTEIN; Lti6b, LOW‐TEMPERATURE‐INDUCED PROTEIN 6B; LRX, LEUCINE‐RICH REPEAT EXTENSIN; RALF, RAPID ALKALINIZATION FACTOR; OST2, OPEN STOMATA2; PME, PECTIN METHYL ESTERASE; PMEI, PME‐INHIBITOR; EGCG, Epigallocatechin gallate; ns, not significant; Fluo. Int., Fluorescence intensity; OE, Overexpression.

Additional interactors include the plasma membrane AUTOINHIBITED H+‐ATPase 1 (AHA1) and AHA3 (Figs 4a, S7A,B). Compared with the Lti6b‐GFP control, MS intensity of AHA1 was 2,7‐fold enriched (Fig. 4a), confirming previously published AHA1‐FER interaction in roots (Haruta *et al*., 2014; Du *et al*., 2016). We also found AHA3 to be significantly enriched with peptides only detected in FER‐GFP samples (Figs 4a, S7A,B). Perception of RALF by FER induces phosphorylation of an inhibitory site on AHA1, resulting in apoplastic alkalinization (Pearce *et al*., 2010; Haruta *et al*., 2014). Since some fungal pathogens modify the apoplastic pH for successful infection (Kesten *et al*., 2019), we hypothesized that FER‐RALF‐induced pH modulations may affect powdery mildew infection success. An *aha1* single mutant did not display *Ecr* colonization differences (Weis *et al*., 2013). While this might be explained by genetic redundancy, *aha1 aha2* double mutants are seedling lethal, making genetic analysis difficult (Haruta *et al*., 2010).

Phosphorylation of the C‐terminal penultimate threonine residue in AHA1/AHA2 (AHA1^T947^/AHA2^T948^) activates AHA proton pump activity (Falhof *et al*., 2016). We tested whether *Ecr* infection affects AHA1^T947^/AHA2^T948^ phosphorylation by using a phosphosite‐specific antibody. However, pT947/pT948 phosphorylation was not altered upon *Ecr* infection (Fig. S9A,B). RALF perception by FER promotes phosphorylation of serine 899 (pS899) to inhibit AHA1/AHA2 proton pump activity (Haruta *et al*., 2014; Liu *et al*., 2025; Sun *et al*., 2025). Importantly, pS899 does not correlate with reduced pT947/pT948 and RALF23/RALF33 treatment does not affect pT947/pT948 (Haruta *et al*., 2014; Liu *et al*., 2025). Therefore, the results do not exclude a FER‐dependent regulation of AHA activity correlated with altered *Ecr* infection success. To investigate a potential role of AHA1 in *Ecr* susceptibility, we tested the *ost2‐2D* mutant producing a constitutive active AHA1 (Merlot *et al*., 2007). The *ost2‐2D* mutant displayed enhanced *Ecr* resistance (Fig. 4b). Interestingly, the related Ler *ost2‐1D* mutant displays reduced PTI responses, similar to *fer*, thus uncoupling PTI efficiency from *Ecr* infection success (Stegmann *et al*., 2017; Zhai *et al*., 2026). The fungal toxin fusicoccin induces constitutive AHA activation and promotes powdery mildew resistance in barley (Baunsgaard *et al*., 1998; Zhou *et al*., 2000). Indeed, fusicoccin treatment could also induce *Ecr* resistance and promote the *fer‐4* resistance phenotype (Fig. 4c,d).

We hypothesized that alkaline pH infiltration may enhance susceptibility and/or break the *fer*‐*4* resistance phenotype, whereas acidic buffers may promote resistance. We infiltrated MES buffer at pH 5.5 and pH 6.5 into the leaf apoplast before infection. Infiltration of a 15 mM MgCl_2_ solution (pH 7) served as an osmolarity control. Surprisingly, both infiltration of the more acidic and more alkaline buffer reduced conidiophore production in comparison with the controls, both in Col‐0 and *fer*‐4 (Fig. 4e,f). We next tested a more alkaline buffer and infiltrated pH 7.5 buffered HEPES into the apoplast before *Ecr* infection. In line with pH 5.5 and pH 7.5 buffered MES, pH 7.5 buffered HEPES induced strong *Ecr* resistance in both Col‐0 and *fer‐4* (Fig. S9C). When analyzing individual fungal colonies upon infection on Col‐0 MES pH 5.5 prebuffered leaves we observed a morphological similarity to fungal colonies on *fer*‐*4* (Fig. 4f). This observation suggests the importance of apoplastic pH modulation for successful conidiophore production of *Ecr* at later time points of infection, which may be regulated through FER‐RALF‐dependent signaling.

Among other physiological outputs, apoplastic pH is involved in growth regulation by controlling PME activity (Hocq *et al*., 2016, 2023; Sénéchal *et al*., 2017). PMEs de‐esterify cell wall pectins, which increases the negative charge of homogalacturonans resulting in enhanced Ca^2+^ binding and pectin cross‐linking for cell wall stiffening (Willats *et al*., 2001). Importantly, demethylated pectin interacts with RALFs and LRX‐RALF complexes to structurally organize the cell wall of root hairs and pollen tubes (Moussu *et al*., 2023; Schoenaers *et al*., 2024). Recently, *fer‐4* mutants were shown to have constitutively increased PME activity in seedlings, which we confirmed in leaf tissue (Fig. 4g; Biermann *et al*., 2025). Similarly, PME activity was enhanced in *lrx5x* and CRISPR *ralf7x*. *Ecr* infection, however, had no effect on PME activity in the harvested leaf samples (Fig. 4g). In accordance with previous observations in the hypocotyl (Biermann *et al*., 2025), leaf staining with the fluorescently labelled chitosan oligosaccharide probe (COS‐488), which binds to de‐esterified pectin oligomers (Mravec *et al*., 2017), displayed higher signals in *fer‐4* compared with Col‐0 (Fig. 4h,i). Unfortunately, we could not analyze changes in the pectin methylesterification status around *Ecr* infection sites because of strong COS‐488 staining of fungal structures (Fig. S9D). To test whether PME activity affects *Ecr* infection, we infiltrated leaves with EGCG that inhibits PMEs (Lewis *et al*., 2008). EGCG pretreatment promoted *Ecr* resistance on Col‐0 but had no effect in *fer‐4* (Fig. 4j,k). To complement the inhibitor treatment, we tested the effect of PME‐INHIBITOR 3 (PMEI3) overexpression on *Ecr* colonization. Overexpression of *PMEI3* inhibits RALF1 signaling in roots (Rößling *et al*., 2024). *PMEI3* overexpression resulted in reduced fungal colonization (Fig. 4l). We also tested *pme3* and *pme17* mutants because of their role in *B. cinerea* infection and FER‐dependent epidermal cell lobing (Raiola *et al*., 2011; Del Corpo *et al*., 2020; W. Tang *et al*., 2022). Knockout of single *PMEs*, however, had no effect on conidiophore production (Fig. S9E). Taken together, full PME activity, which affects FER‐dependent RALF signaling, is required for powdery mildew susceptibility.

### RALF‐mediated powdery mildew susceptibility is partially independent of FER and FER signaling

**Fig. 5:**
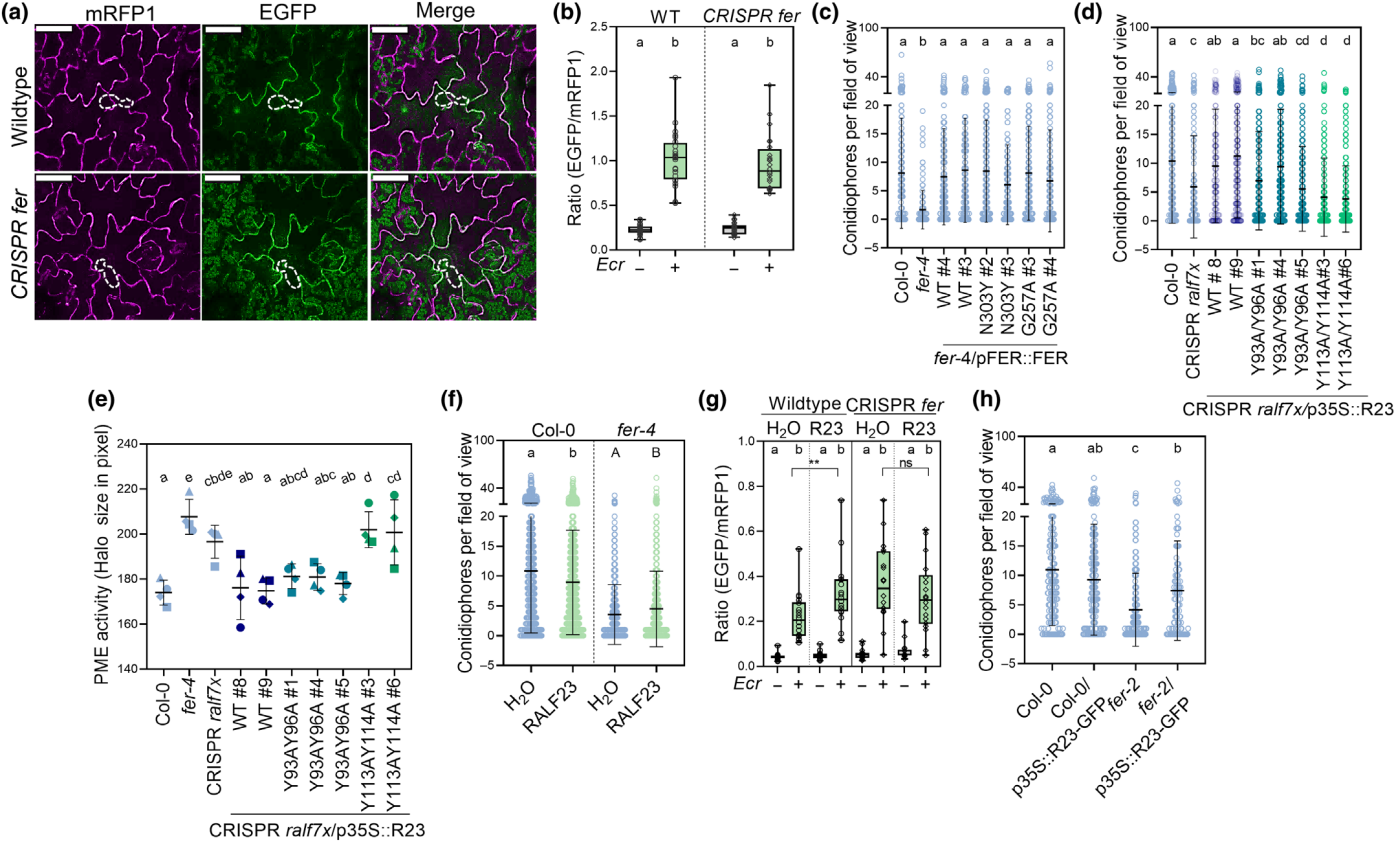
RALF‐mediated powdery mildew susceptibility of Arabidopsis is partially independent of FER and FER signaling. (a) Confocal images of pUBQ10::SYP122‐pHusion lines in the Col‐0 and CRISPR *fer* background (2 dpi). Dotted lines indicate the location of the fungal spore. Bars, 50 μm. (b) Quantification of the EGFP/mRFP1 ratio near the penetration site (green) and in uninfected areas (gray). Mean ± SD, *n* = 24 pooled from three independent experiments (Mann–Whitney test, a‐b *P* < 0.0001). Boxes represent the interquartile range (IQR) with the center line indicating the median; whiskers extend from minimum to maximum values; all data points are shown. (c) Conidiophores per field of view (5 dpi) of fungal colonies grown upon *Ecr* infection of the indicated genotypes. Mean ± SD, *n* = 131–210 pooled from four independent experiments (Dunn's multiple comparisons test, a‐b *P* < 0.0001). (d) Conidiophores per field of view (5 dpi) of fungal colonies grown upon *Ecr* infection of the indicated genotypes. Mean ± SD, *n* = 168–326 pooled from six independent experiments (Dunn's multiple comparisons test, a‐bc *P* = 0.0469, a‐cd *P* < 0.0001, a‐d *P* < 0.0001, ab‐cc *P* ≤ 0.0004, bc‐dc *P* ≤ 0.0019). (e) PME activity normalized to Col‐0. Mean ± SD, *n* = 4, data points with different symbols indicate independent biological replicates (Tukey's multiple comparisons test, a‐e *P* ≤ 0.0003, a‐d *P* ≤ 0.0034, a‐cd *P* = 0.0058, ab‐e ≤ 0.0012, ab‐d ≤ 0.0138, ab‐cd≤0.0227, a‐cbde≤0.0326). (f) Conidiophores per field of view (5 dpi) after pretreatment with H_2_O (blue) or 1 μM RALF23 before *Ecr* infection. Mean ± SD, *n* = 485–970 pooled from 11 independent experiments (Mann–Whitney test, statistical analysis was performed on each genotype individually, Col‐0: a‐b *P* = 0.0008, *fer‐4:* A‐B *P* = 0.0064). (g) Quantification of the EGFP/mRFP1 ratio near the penetration site (green) and in uninfected areas (gray) upon *Ecr* infection at 2 dpi. One micrometer synthetic RALF peptide or H_2_O was infiltrated 2 h before infection. Mean ± SD, *n* = 18 pooled from three independent experiments (Dunn's multiple comparisons test a‐b *P* ≤ 0.0412, Mann–Whitney test, **P = 0.0064, ns = not significant). Boxes represent the interquartile range (IQR) with the center line indicating the median; whiskers extend from minimum to maximum values; all data points are shown. (h) Conidiophores per field of view (5 dpi) of fungal colonies grown upon *Ecr* infection of the indicated genotypes. Mean ± SD, *n* = 95–137 pooled from three independent experiments (Dunn's multiple comparisons test, a‐b *P* = 0.0076, a‐c *P* < 0.0001, b‐c *P* = 0.0017). All experiments were performed at least three times with similar results. Dpi, days post infection; *Ecr, Erysiphe cruciferarum*; EGFP, enhanced GREEN FLUORESCENCE PROTEIN; FER, FERONIA; PME, PECTIN METHYL ESTERASE; RALF, RAPID ALKALINIZATION FACTOR; RFP, RED FLUORESCENCE PROTEIN; R23, RALF23.

The reduced *Ecr* infection success upon pH buffering prompted us to resolve potential infection‐induced apoplastic pH changes with cellular resolution with the ratio‐metric pH sensor pUBQ10::SYP122‐pHusion (Kesten *et al*., 2019). We detected apoplastic pH increases close to *Ecr* penetration sites at 2 dpi. Surprisingly, infection‐related pH increases were also observed in pUBQ10::SYP122‐pHusion CRISPR *fer* (Figs 5a,b, S10A).

To assess whether FER recruitment to RALF‐LLG1 complexes is required for *Ecr* susceptibility, we infected *fer*‐*4* lines expressing structure‐guided FER mutants disrupting the LLG binding interface (Xiao *et al*., 2019). Both FER^N303Y^ and FER^G257A^ restored powdery mildew susceptibility, indicating that RALF‐induced recruitment of FER to LLG1 is not essential for *Ecr* infection (Fig. 5c). We thus asked the question whether the susceptibility function of RALFs is linked to cell wall structural functions. We introduced mutations into RALF23, either disrupting the predicted LRX‐binding interface (RALF23^Y113A/Y114A^), analogous to RALF4^Y83A/Y84A^ (Moussu *et al*., 2020) or the YISY motif required for LLG‐FER‐dependent signaling (RALF23^Y93A/Y96A^; Xiao *et al*., 2019). We expressed both variants under control of the 35S promoter in CRISPR *ralf7x*. We tested two and three lines, respectively, with CRISPR *ralf7x* p35S::RALF23^Y93A/Y96A^ displaying similar expression levels as the WT complementation lines. CRISPR *ralf7x* p35S::RALF23^Y113A/Y114A^ lines showed weaker expression compared with the WT construct (Fig. S3F,G). RALF23^Y113A/Y114A^ could not complement the CRISPR *ralf7x* growth and enhanced *Ecr* resistance phenotype (Figs 5d, S2). RALF23^Y93A/Y96A^ largely complemented the growth phenotype (Fig. S2), but only one out of three tested lines fully restored *Ecr* susceptibility (Fig. 5d). We also generated pRALF23 (pR23)::mCherry‐RALF23 lines, with the mCherry tag located between the signal peptide and RALF23, carrying the same mutations. All RALF variants marked the outline of epidermal cells (Fig. S11, Panel 1). Upon plasmolysis, a strong protoplastic signal for all mCherry‐RALF23 variants was detected (Fig. S11, Panel 2). However, mCherry‐RALF23^WT^ and mCherry‐RALF23^Y93A/Y96A^ displayed strong remaining signal at the cell wall, while mCherry‐RALF23^Y113A/Y114A^ was fully depleted (Fig. S11, Panels 1–4). This suggests loss of cell wall binding in RALF23^Y113A/Y114A^ (Fig. S11). RALF23 cell wall localization is consistent with previous reports on RALF22 and RALF4 (Moussu *et al*., 2023; Schoenaers *et al*., 2024). RALF22 secretion is impaired in *lrx1/2* (Schoenaers *et al*., 2024). By contrast, RALF23 secretion is neither affected by disrupting LRX nor LLG1 binding motives. RALF23 and RALF23^Y93A/Y96A^, but not RALF23^Y113A/Y114A^, could rescue enhanced PME activity of CRISPR *ralf7x* (Fig. 5e), suggesting that RALF23 interaction with LRXs is essential to inhibit PME function. Together, these data implicate that both interaction of RALF23 with LLG and LRX complexes is required to support full *Ecr* colonization with the latter playing a predominant role for RALF‐regulated PME activity and powdery mildew susceptibility.

We next tested whether application of synthetic RALF23 peptide can affect powdery mildew infection success. Surprisingly, infiltration of 1 μM RALF23 into single leaves 24 h before *Ecr* infection had a mild resistance‐inducing effect in Col‐0 (Fig. 5f), while the infiltration of lower concentrations (10 nM and 100 nM) had no significant effect (Fig. S10B). Remarkably, RALF23 infiltration increased conidiophore production in the *fer‐4* mutant background, suggesting that RALF23 exerts FER‐independent effects (Fig. 5f). Interestingly, infiltration with RALF23 promoted alkalinization near the infection site in pUBQ10::SYP122‐pHusion, but not in CRISPR *fer* (Fig. 5g). Surprisingly, water infiltration (control) increased *Ecr*‐induced alkalinization in CRISPR *fer*. This raises the question whether FER is additionally involved in buffering *Ecr*‐induced pH changes upon apoplast infiltration. Also, RALF23 infiltration promoted GFP fluorescence in pUBQ::SYP122‐pHusion in a FER‐dependent manner at early time points post treatment. Buffering abolished the recorded pH shifts (Fig. S10C), suggesting that it effectively compromises RALF23 pH changes. We continued testing the effect of RALF23‐GFP overexpression on *Ecr* infection outcome. Similar to exogenous RALF23 treatment (Fig. 5f), RALF23‐GFP overexpression displayed a trend toward enhanced *Ecr* resistance in Col‐0 (Fig. 5h). In *fer‐2*, RALF23‐GFP overexpression promoted susceptibility (Fig. 5h) and displayed a clear trend to reduce the mutant's enhanced PME activity (Fig. S10D). This observation is in line with previous findings that RALF23 treatment significantly reduces PME activity in *fer‐4*, but not in Col‐0 (Biermann *et al*., 2025). *RALF23‐GFP* showed overexpression in both Col‐0 and *fer‐2* (Fig. S10D).

Collectively, our data suggest that RALF‐mediated powdery mildew susceptibility is at least partially FER‐independent. Moreover, it indicates that the likely appropriate spatio‐temporal availability of RALFs affects *Ecr* reproductive success in a combination of partially FER‐dependent effects on alkalinization and FER‐independent responses, potentially related to RALF‐regulated PME activity.

## Discussion

This work provides first molecular insights into the mechanism of FER‐mediated powdery mildew susceptibility. We revealed that FER's RALF peptide ligands are critical for the completion of *Ecr's* asexual life cycle upon host colonization.

RALFs are perceived by LLG‐FER/CrRLK1L complexes to induce downstream signaling and bind to cell wall localized LRX proteins and demethylated pectin for cell wall organization. Our data suggest that powdery mildew sporulation requires functional apoplastic pH homeostasis and modifications of the cell wall pectin methyl‐esterification status, both processes in which FER‐RALF and/or LRX‐RALF modules fulfill central regulatory functions. Earlier work revealed that barley powdery mildew infection is accompanied by pH shifts and the induction of apoplastic alkalinization at early time points post inoculation (2 h; Felle *et al*., 2004). Importantly though, mildew‐induced pH shifts were dynamic, with fluctuations between alkaline and more acidic conditions during the recorded time period (50 h; Felle *et al*., 2004). In light of our observations, disrupting apoplastic pH homeostasis by buffering, RALF23 peptide treatments or RALF23 overexpression may circumvent dynamic endogenous regulatory circuits and inhibit *Ecr* host colonization. A challenge for the future will be to reveal spatio‐temporal pH fluctuations and RALF secretion during powdery mildew infection.

In addition, the pectin methyl‐esterification status contributes to powdery mildew pathogenesis. Both EGCG treatment and PMEI3 overexpression promoted *Ecr* resistance. Notably, RALF‐signaling outputs and the methyl‐esterification status of the cell wall are tightly interconnected. PMEI3 is strongly pH‐dependent with the highest activity at acidic conditions (Xu *et al*., 2022). RALF‐induced apoplastic alkalinization may inhibit PMEI3, which increases PME activity for cell wall remodeling. RALF22 and RALF4 bind to LRXs to affect pectin compaction in the cell wall (Moussu *et al*., 2023; Schoenaers *et al*., 2024). In return, PME activity modulates RALF1/RALF23 signaling and the formation of RALF1‐pectin molecular condensates required for FER‐LLG1‐dependent responses (Liu *et al*., 2024; Rößling *et al*., 2024; Biermann *et al*., 2025). Consistently, EGCG treatment enhanced powdery mildew resistance in Col‐0 but had no additive effect in *fer‐4*. Considering that *fer‐4* is strongly compromised in RALF perception, our data indicate that PME inhibition through EGCG treatment affects the same FER‐dependent pathway. We propose that EGCG treatment and PMEI3 overexpression block sustained *Ecr* growth by reducing the availability of demethylated pectin for RALF signaling through FER. Fusicoccin and buffer treatment could also induce resistance in *fer‐4*, suggesting additional effects that do not solely rely on pH‐dependent PME regulation. Our work revealed that *fer*, CRISPR *ralf7x* and *lrx5x* display elevated steady‐state PME activity, in accordance with previous findings (Biermann *et al*., 2025). Moreover, PME activity is primarily regulated by RALF binding to LRXs. This raises the question of whether disruption of the FER‐RALF‐LRX pathway activates feedback mechanisms to promote PME activity in an effort to re‐establish RALF cell wall binding and signaling. Interestingly, RALF23 treatment and overexpression in *fer* can reduce PME activity (Biermann *et al*., 2025) and enhance powdery mildew susceptibility. This indicates FER‐independent effects of RALF23 on cell wall organization and powdery mildew infection. FER is dispensable for RALF22‐induced root hair tip alkalinization, calcium influx and cell wall modification (Schoenaers *et al*., 2024), and RALF23 affects cell wall pectin status in a partially FER‐independent and LRX‐dependent manner (Biermann *et al*., 2025). Our SYP122‐pHusion results suggest that *Ecr* induces apoplastic pH shifts independent of FER, raising the question of receptor redundancy or RALF‐independent pH modulations, possibly through PTI (Wang *et al*., 2026; Zhai *et al*., 2026) or, alternatively, actively triggered by the fungus. Interestingly, though, RALF23‐induced enhancement of *Ecr*‐triggered apoplastic pH modulation is FER‐dependent. This is in line with our findings that RALF23 interaction with FER‐LLG1 supports full colonization. However, RALF23 binding to LRXs is important for cell wall binding and essential for *Ecr* infection success. This suggests a predominant role for RALF23 structural function and/or effects on cell wall chemistry to support *Ecr* colonization. Yet, the interplay between RALF structural and signaling function remains largely unknown (Schade *et al*., 2025). RALFs bind to LRX with high affinity at acidic pH, while LLGs require alkaline conditions for optimal RALF recognition (Moussu *et al*., 2020). It is possible that dynamic apoplastic pH fluctuations induced by *Ecr* infection release RALFs from LRXs to trigger CrRLK1L‐dependent signaling that further affects its reproductive success. Our data also raise the question of whether additional FER‐related receptors are required for RALF perception to establish full *Ecr* susceptibility. FER can interact with related RALF‐binding CrRLK1Ls, including HERK1 and ANJ, to form higher order complexes (Galindo‐Trigo *et al*., 2020; Zhong *et al*., 2022; Lan *et al*., 2023). This is supported by our Co‐IP‐MS data, according to which FER interacted with several members of the CrRLK1L family, namely HERK1, MDS1, THE1 and LET1.

The fungal haustorium is surrounded by the extrahaustorial matrix (EHMx) and the extrahaustorial membrane (EHM), a plant derived membrane that is continuous with the plasma membrane but has distinctive properties (Hückelhoven & Panstruga, 2011; Polonio *et al*., 2021). At the site of invasion the EHMx is sealed with densely packed cell wall depositions forming the haustorial neck, rendering the EHMx a separate apoplastic compartment (Micali *et al*., 2011). Nutrients taken up from the EHMx should derive from the invaded plant cell and require prior export. Plant cell sugar uptake is facilitated by SUGAR TRANSPORT PROTEINs (STP), which are associated with plant microbe interactions (Bezrutczyk *et al*., 2018). In Arabidopsis, STP8‐GFP, upon overexpression, localizes to the EHM and increases susceptibility to powdery mildew (Liu *et al*., 2021) and transport‐compromised STP13 alleles are associated with resistance to powdery mildew in wheat and barley (Milne *et al*., 2019). STPs are H^+^ symporters, and STP8 has a pH optimum at 5.5 for cellular import (Liu *et al*., 2021). It is possible that *Ecr* infection induces RALF‐CrRLK1L‐dependent changes to the apoplastic pH that reprograms transporter topology to favor haustorial sugar uptake over plant cell import. In accordance, *fer* mutants show altered sucrose uptake and starch accumulation (Yang *et al*., 2015; Yeats *et al*., 2016). Supporting our hypothesis, we identified several transporter proteins as FER interactors in our Co‐IP‐MS experiment, including STP1, involved in resistance against bacterial infection (Yamada *et al*., 2016). We did not identify many differential FER‐GFP interactors upon *Ecr* infection, likely due to the early experimental time point chosen (1 dpi).

**Fig. 6:**
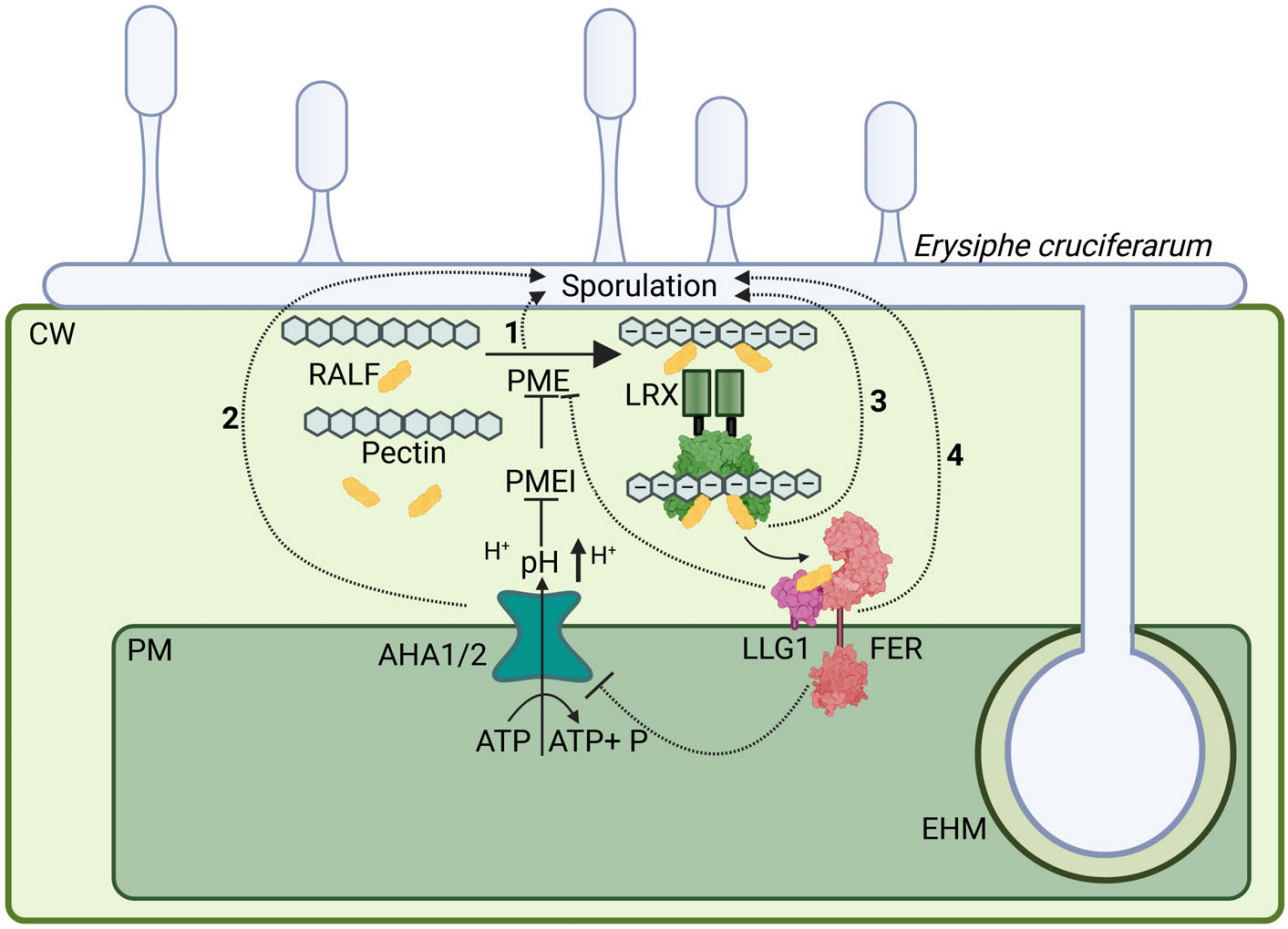
Model of RALF‐FER‐dependent effects on powdery mildew sporulation. RALF peptides bind to negatively charged pectins and LRXs in the cell wall, as well as the FER‐LLG1 complex at the plasma membrane. RALF binding to FER‐LLG1 results in inhibition of AHA1/2 activity and apoplastic pH changes. The apoplastic pH modulates PMEI/PME activity and with this likely the methyl‐esterification status of cell wall pectins. Both PME activity (1) and apoplastic pH modulation (2) affect powdery mildew sporulation. RALF peptides appear to affect sporulation in a FER‐independent (3) and FER‐dependent (4) manner, potentially through regulating cell wall structure and the apoplastic pH. AHA, AUTOINHIBITED H + ‐ATPase; ATP, Adenosine triphosphate; CW, cell wall; EHM, Extrahaustorial membrane; FER, FERONIA; GFP, GREEN FLUORESCENCE PROTEIN; LLG1, LORELEI‐LIKE GPI‐ANCHORED PROTEIN 1, LRX, LEUCINE‐RICH REPEAT EXTENSIN; PM, Plasma membrane; PME, PECTIN METHYL ESTERASE; PMEI, PME‐INHIBITOR; RALF, RAPID ALKALINIZATION FACTOR. This figure was created in BioRender (Biorender.com; Leicher, H. (2026) https://BioRender.com/ksausa0. Arrow: positive interaction, blunt‐ended arrow: negative interaction. Dashed line: hypothesized pathway.

We propose a model in which RALF‐mediated apoplastic pH modulation and pectin de‐methylesterification are a prerequisite for sustained powdery mildew host colonization (Fig. 6). In accordance, endogenous pectin modification affects powdery mildew infection outcome. Mutants in the pectate lyase *POWDERY MILDEW RESISTANT 6* (*PMR6*) and the pectin acetyltransferase *PMR5* are more resistant to powdery mildew without altered fungal penetration or deregulated SA/JA signaling (Vogel *et al*., 2002, 2004). Our data show that RALF pathway mutants display a similar phenotype. Moreover, the *fer‐4* mutant has reduced cell wall cellulose content (Yeats *et al*., 2016) and dysfunctional apoplastic pH modulation, which could interfere with fungal nutrient uptake. This supports a previously coined hypothesis of altered fungal nutrition as a consequence of cell wall defects (Vogel *et al*., 2002, 2004).

Other plant pathogens produce RALFs to establish host colonization, including *F. oxysporum* and root knot nematodes (Masachis *et al*., 2016; Zhang *et al*., 2020). Interestingly, these RALF peptide mimics lack conserved sequence elements required for LRX binding but contain a modified YISY motif required for LLG‐CrRLK1L‐dependent signaling (Pearce *et al*., 2010). This suggests that fungal RALFs support virulence mainly through CrRLK1L‐induced signaling and less through cell wall binding. None of the analyzed powdery mildew genomes contained RALF peptide mimics, suggesting that *Ecr* likely relies on the combination of endogenous RALF signaling and cell wall remodeling to support sustained host interaction. Our work thus highlights a new mode of RALF function, combining signaling and structural roles for the control of plant microbe interactions.

## Competing interests

None declared.

## Author contributions

MS contributed to the conceptualization. HL, SDS, JWH, KSM‐E, GH, DB, AM, XZ, YC, MCM, AB‐D and MS contributed to the investigation. MS, RH, AB‐D, MKR‐L, JG, TE, TK, MCM and CL contributed to the funding acquisition. MS contributed to the project administration. MS, HL, RH, AB‐D, MKR‐L, JG, TE and CL contributed to the supervision. HL and MS contributed to the writing – original draft. All authors contributed to the writing – review and editing.

## Disclaimer

The New Phytologist Foundation remains neutral with regard to jurisdictional claims in maps and in any institutional affiliations.

## Supporting information


**Fig. S1** Quantification of fungal growth on mutants of RALF binding proteins.
**Fig. S2** Images of plant lines used in this study.
**Fig. S3** Characterization of CRISPR *ralf* mutants.
**Fig. S4** Regulation of RALF peptides during powdery mildew infection.
**Fig. S5** FER‐ and RALF‐mediated powdery mildew susceptibility is most likely not directly linked to MLO.
**Fig. S6** Characterization of *fer‐4* CRISPR *myc2*.
**Fig. S7** FER interactors detected by Co‐IP‐MS.
**Fig. S8** Powdery mildew dependent FER interactors detected by Co‐IP‐MS.
**Fig. S9** Role of apoplastic pH and PMEs for *Ecr* infection success.
**Fig. S10** Analysis of FER‐independent RALF23 functions during *Ecr* infection.
**Fig. S11** Cell wall localization of mCherry‐RALF23 peptide variants.
**Table S1** Primers used in this study.
**Table S2** CRISPR target sites.
**Table S3** Plant lines used in this study.
**Table S4** Identification of RALF‐like peptides from phytopathogenic fungi species reported to be able to infect Arabidopsis.
Please note: Wiley is not responsible for the content or functionality of any Supporting Information supplied by the authors. Any queries (other than missing material) should be directed to the *New Phytologist* Central Office.

## Data Availability

The mass spectrometric raw files as well as the MaxQuant output files have been deposited to the ProteomeXchange Consortium via the PRIDE partner repository and can be accessed using the identifier PXD059737 (https://proteomecentral.proteomexchange.org/cgi/GetDataset?ID=PXD059737). Source data (numeric data displayed in figures, raw images of microscopy/western blots) are available from Zenodo (https://zenodo.org/records/20322490).

## References

[nph71282-bib-0001] Abarca A , Franck CM , Zipfel C . 2021. Family‐wide evaluation of RAPID ALKALINIZATION FACTOR peptides. Plant Physiology 187: 996–1010. 34608971 10.1093/plphys/kiab308 PMC8491022

[nph71282-bib-0002] Acevedo‐Garcia J , Spencer D , Thieron H , Reinstädler A , Hammond‐Kosack K , Phillips AL , Panstruga R . 2017. mlo‐based powdery mildew resistance in hexaploid bread wheat generated by a non‐transgenic TILLING approach. Plant Biotechnology Journal 15: 367–378. 27565953 10.1111/pbi.12631 PMC5316926

[nph71282-bib-0003] Asai S , Shirasu K , Jones JDG . 2015. *Hyaloperonospora arabidopsidis* (Downy Mildew) infection assay in Arabidopsis. Bio‐Protocol 5: e1627.

[nph71282-bib-0004] Baunsgaard L , Fuglsang AT , Jahn T , Korthout HAAJ , De Boer AH , Palmgren MG . 1998. The 14–3‐3 proteins associate with the plant plasma membrane H1‐ATPase to generate a fusicoccin binding complex and a fusicoccin responsive system. The Plant Journal 13: 661–671. 9681008 10.1046/j.1365-313x.1998.00083.x

[nph71282-bib-0005] Bethke G , Grundman RE , Sreekanta S , Truman W , Katagiri F , Glazebrook J . 2014. Arabidopsis PECTIN METHYLESTERASEs contribute to immunity against Pseudomonas syringae. Plant Physiology 164: 1093–1107. 24367018 10.1104/pp.113.227637 PMC3912082

[nph71282-bib-0006] Bezrutczyk M , Yang J , Eom J‐S , Prior M , Sosso D , Hartwig T , Szurek B , Oliva R , Vera‐Cruz C , White FF *et al*. 2018. Sugar flux and signaling in plant–microbe interactions. The Plant Journal 93: 675–685. 29160592 10.1111/tpj.13775

[nph71282-bib-0007] Bhat RA , Miklis M , Schmelzer E , Schulze‐Lefert P , Panstruga R . 2005. Recruitment and interaction dynamics of plant penetration resistance components in a plasma membrane microdomain. Proceedings of the National Academy of Sciences, USA 102: 3135–3140. 10.1073/pnas.0500012102 PMC549507 15703292

[nph71282-bib-0008] Biermann D , von Arx M , Munzert‐Eberlein KS , Xhelilaj K , Séré D , Stegmann M , Vert G , Wolf S , Engelsdorf T , Zipfel C *et al*. 2025. A RALF‐brassinosteroid signaling circuit regulates Arabidopsis hypocotyl cell shape. Current Biology 35: 5002–5017. 41015041 10.1016/j.cub.2025.09.016

[nph71282-bib-0009] Bürger M , Chory J . 2019. Stressed out about hormones: How plants orchestrate immunity. Cell Host & Microbe 26: 163–172. 31415749 10.1016/j.chom.2019.07.006 PMC7228804

[nph71282-bib-0010] Büschges R , Hollricher K , Panstruga R , Simons G , Wolter M , Frijters A , van Daelen R , van der Lee T , Diergaarde P , Groenendijk J *et al*. 1997. The Barley *Mlo* gene: a novel control element of plant pathogen resistance. Cell 88: 695–705. 9054509 10.1016/s0092-8674(00)81912-1

[nph71282-bib-0011] Castel B , Tomlinson L , Locci F , Yang Y , Jones JDG . 2019. Optimization of T‐DNA architecture for Cas9‐mediated mutagenesis in Arabidopsis. PLoS ONE 14: e0204778. 30625150 10.1371/journal.pone.0204778 PMC6326418

[nph71282-bib-0012] Consonni C , Humphry ME , Hartmann HA , Livaja M , Durner J , Westphal L , Vogel J , Lipka V , Kemmerling B , Schulze‐Lefert P *et al*. 2006. Conserved requirement for a plant host cell protein in powdery mildew pathogenesis. Nature Genetics 38: 716–720. 16732289 10.1038/ng1806

[nph71282-bib-0013] Cox J , Hein MY , Luber CA , Paron I , Nagaraj N , Mann M . 2014. Accurate proteome‐wide label‐free quantification by delayed normalization and maximal peptide ratio extraction, termed MaxLFQ*. Molecular & Cellular Proteomics 13: 2513–2526. 24942700 10.1074/mcp.M113.031591 PMC4159666

[nph71282-bib-0014] Del Corpo D , Fullone MR , Miele R , Lafond M , Pontiggia D , Grisel S , Kieffer‐Jaquinod S , Giardina T , Bellincampi D , Lionetti V . 2020. AtPME17 is a functional Arabidopsis thaliana pectin methylesterase regulated by its PRO region that triggers PME activity in the resistance to *Botrytis cinerea* . Molecular Plant Pathology 21: 1620–1633. 33029918 10.1111/mpp.13002 PMC7694680

[nph71282-bib-0015] Du C , Li X , Chen J , Chen W , Li B , Li C , Wang L , Li J , Zhao X , Lin J *et al*. 2016. Receptor kinase complex transmits RALF peptide signal to inhibit root growth in Arabidopsis. Proceedings of the National Academy of Sciences, USA 113: E8326–E8334. 10.1073/pnas.1609626113 PMC5187724 27930296

[nph71282-bib-0016] Duan Q , Kita D , Li C , Cheung AY , Wu H‐M . 2010. FERONIA receptor‐like kinase regulates RHO GTPase signaling of root hair development. Proceedings of the National Academy of Sciences, USA 107: 17821–17826. 10.1073/pnas.1005366107 PMC2955125 20876100

[nph71282-bib-0017] Dünser K , Gupta S , Herger A , Feraru MI , Ringli C , Kleine‐Vehn J . 2019. Extracellular matrix sensing by FERONIA and Leucine Rich Repeat Extensins controls vacuolar expansion during cellular elongation in *Arabidopsis thaliana* . EMBO Journal 38: e100353. 30850388 10.15252/embj.2018100353 PMC6443208

[nph71282-bib-0018] Engelhardt S , Stam R , Hückelhoven R . 2018. Good riddance? Breaking disease susceptibility in the era of new breeding technologies. Agronomy 8: 114.

[nph71282-bib-0019] Engelsdorf T , Gigli‐Bisceglia N , Veerabagu M , McKenna JF , Vaahtera L , Augstein F , Van der Does D , Zipfel C , Hamann T . 2018. The plant cell wall integrity maintenance and immune signaling systems cooperate to control stress responses in *Arabidopsis thaliana* . Science Signaling 11: eaao3070. 29945884 10.1126/scisignal.aao3070

[nph71282-bib-0020] Falhof J , Pedersen JT , Fuglsang AT , Palmgren M . 2016. Plasma membrane H+‐ATPase regulation in the center of plant physiology. Molecular Plant 9: 323–337. 26584714 10.1016/j.molp.2015.11.002

[nph71282-bib-0021] Felle HH , Herrmann A , Hanstein S , Hückelhoven R , Kogel K‐H . 2004. Apoplastic pH signaling in barley leaves attacked by the powdery mildew fungus *Blumeria graminis* f. sp. hordei. Molecular Plant Microbe Interactions 17: 118–123. 14714875 10.1094/MPMI.2004.17.1.118

[nph71282-bib-0022] Galindo‐Trigo S , Blanco‐Touriñán N , DeFalco TA , Wells ES , Gray JE , Zipfel C , Smith LM . 2020. *Cr*RLK1L receptor‐like kinases HERK1 and ANJEA are female determinants of pollen tube reception. EMBO Reports 21: e48466. 31867824 10.15252/embr.201948466 PMC7001495

[nph71282-bib-0023] Gao Q , Wang C , Xi Y , Shao Q , Li L , Luan S . 2022. A receptor–channel trio conducts Ca^2+^ signalling for pollen tube reception. Nature 607: 534–539. 35794475 10.1038/s41586-022-04923-7 PMC9308748

[nph71282-bib-0024] Ge Z , Bergonci T , Zhao Y , Zou Y , Du S , Liu M‐C , Luo X , Ruan H , García‐Valencia LE , Zhong S *et al*. 2017. Arabidopsis pollen tube integrity and sperm release are regulated by RALF‐mediated signaling. Science 358: 1596–1600. 29242234 10.1126/science.aao3642 PMC5964610

[nph71282-bib-0025] Glawe DA . 2008. The powdery mildews: a review of the world's most familiar (yet poorly known) plant pathogens. Annual Review of Phytopathology 46: 27–51. 10.1146/annurev.phyto.46.081407.104740 18680422

[nph71282-bib-0026] Gonneau M , Desprez T , Martin M , Doblas VG , Bacete L , Miart F , Sormani R , Hématy K , Renou J , Landrein B *et al*. 2018. Receptor kinase THESEUS1 is a rapid alkalinization factor 34 receptor in Arabidopsis. Current Biology 28: 2452–2458. 30057301 10.1016/j.cub.2018.05.075

[nph71282-bib-0027] Gronnier J , Franck CM , Stegmann M , DeFalco TA , Abarca A , von Arx M , Dünser K , Lin W , Yang Z , Kleine‐Vehn J *et al*. 2022. Regulation of immune receptor kinase plasma membrane nanoscale organization by a plant peptide hormone and its receptors. eLife 11: e74162. 34989334 10.7554/eLife.74162 PMC8791635

[nph71282-bib-0028] Guo H , Nolan TM , Song G , Liu S , Xie Z , Chen J , Schnable PS , Walley JW , Yin Y . 2018. FERONIA receptor kinase contributes to plant immunity by suppressing jasmonic acid signaling in *Arabidopsis thaliana* . Current Biology 28: 3316–3324.e3316. 30270181 10.1016/j.cub.2018.07.078

[nph71282-bib-0029] Haruta M , Burch HL , Nelson RB , Barrett‐Wilt G , Kline KG , Mohsin SB , Young JC , Otegui MS , Sussman MR . 2010. Molecular characterization of mutant Arabidopsis plants with reduced plasma membrane proton pump activity. Journal of Biological Chemistry 285: 17918–17929. 20348108 10.1074/jbc.M110.101733 PMC2878554

[nph71282-bib-0030] Haruta M , Sabat G , Stecker K , Minkoff BB , Sussman MR . 2014. A peptide hormone and its receptor protein kinase regulate plant cell expansion. Science 343: 408–411. 24458638 10.1126/science.1244454 PMC4672726

[nph71282-bib-0031] Hayashi Y , Nakamura S , Takemiya A , Takahashi Y , Shimazaki K‐i , Kinoshita T . 2010. Biochemical characterization of *in vitro* phosphorylation and dephosphorylation of the plasma membrane H+‐ATPase. Plant and Cell Physiology 51: 1186–1196. 20516032 10.1093/pcp/pcq078

[nph71282-bib-0032] Herger A , Gupta S , Kadler G , Franck CM , Boisson‐Dernier A , Ringli C . 2020. Overlapping functions and protein‐protein interactions of LRR‐extensins in Arabidopsis. PLoS Genetics 16: e1008847. 32559234 10.1371/journal.pgen.1008847 PMC7357788

[nph71282-bib-0033] Hocq L , Habrylo O , Sénéchal F , Voxeur A , Pau‐Roblot C , Safran J , Fournet F , Bassard S , Battu V , Demailly H *et al*. 2023. Mutation of AtPME2, a pH‐dependent pectin methylesterase, affects cell wall structure and hypocotyl elongation. Plant and Cell Physiology 65: 301–318. 10.1093/pcp/pcad154 38190549

[nph71282-bib-0034] Hocq L , Sénéchal F , Lefebvre V , Lehner A , Domon J‐M , Mollet J‐C , Dehors J , Pageau K , Marcelo P , Guérineau F *et al*. 2016. Combined experimental and computational approaches reveal distinct pH dependence of pectin methylesterase inhibitors. Plant Physiology 173: 1075–1093. 28034952 10.1104/pp.16.01790 PMC5291010

[nph71282-bib-0035] Hok S , Allasia V , Andrio E , Naessens E , Ribes E , Panabières F , Attard A , Ris N , Clément M , Barlet X *et al*. 2014. The receptor kinase IMPAIRED OOMYCETE SUSCEPTIBILITY1 attenuates abscisic acid responses in Arabidopsis. Plant Physiology 166: 1506–1518. 25274985 10.1104/pp.114.248518 PMC4226379

[nph71282-bib-0036] Hok S , Danchin EGJ , Allasia V , Panabières F , Attard A , Keller H . 2011. An Arabidopsis (malectin‐like) leucine‐rich repeat receptor‐like kinase contributes to downy mildew disease. Plant, Cell & Environment 34: 1944–1957. 10.1111/j.1365-3040.2011.02390.x 21711359

[nph71282-bib-0037] Hückelhoven R , Panstruga R . 2011. Cell biology of the plant–powdery mildew interaction. Current Opinion in Plant Biology 14: 738–746. 21924669 10.1016/j.pbi.2011.08.002

[nph71282-bib-0038] Huebbers JW , Caldarescu GA , Kubátová Z , Sabol P , Levecque SCJ , Kuhn H , Kulich I , Reinstädler A , Büttgen K , Manga‐Robles A *et al*. 2024. Interplay of EXO70 and MLO proteins modulates trichome cell wall composition and susceptibility to powdery mildew. Plant Cell 36: 1007–1035. 38124479 10.1093/plcell/koad319 PMC10980356

[nph71282-bib-0039] Kessler SA , Shimosato‐Asano H , Keinath NF , Wuest SE , Ingram G , Panstruga R , Grossniklaus U . 2010. Conserved molecular components for pollen tube reception and fungal invasion. Science 330: 968–971. 21071669 10.1126/science.1195211

[nph71282-bib-0040] Kesten C , Gámez‐Arjona FM , Menna A , Scholl S , Dora S , Huerta AI , Huang HY , Tintor N , Kinoshita T , Rep M *et al*. 2019. Pathogen induced pH changes regulate the growth defense balance in plants. EMBO Journal 38: e101822. 31736111 10.15252/embj.2019101822 PMC6912046

[nph71282-bib-0041] Kusch S , Panstruga R . 2017. mlo‐based resistance: an apparently universal “Weapon” to defeat powdery mildew disease. Molecular Plant–Microbe Interactions 30: 179–189. 28095124 10.1094/MPMI-12-16-0255-CR

[nph71282-bib-0042] Lan Z , Song Z , Wang Z , Li L , Liu Y , Zhi S , Wang R , Wang J , Li Q , Bleckmann A *et al*. 2023. Antagonistic RALF peptides control an intergeneric hybridization barrier on Brassicaceae stigmas. Cell 186: 4773–4787. 37806310 10.1016/j.cell.2023.09.003 PMC10615786

[nph71282-bib-0043] Lewis KC , Selzer T , Shahar C , Udi Y , Tworowski D , Sagi I . 2008. Inhibition of pectin methyl esterase activity by green tea catechins. Phytochemistry 69: 2586–2592. 18829053 10.1016/j.phytochem.2008.08.012

[nph71282-bib-0044] Li C , Yeh F‐L , Cheung AY , Duan Q , Kita D , Liu M‐C , Maman J , Luu EJ , Wu BW , Gates L *et al*. 2015. Glycosylphosphatidylinositol‐anchored proteins as chaperones and co‐receptors for FERONIA receptor kinase signaling in Arabidopsis. eLife 4: e06587. 26052747 10.7554/eLife.06587 PMC4458842

[nph71282-bib-0045] Liu J , Liu M , Tan L , Huai B , Ma X , Pan Q , Zheng P , Wen Y , Zhang Q , Zhao Q *et al*. 2021. AtSTP8, an endoplasmic reticulum‐localised monosaccharide transporter from Arabidopsis, is recruited to the extrahaustorial membrane during powdery mildew infection. New Phytologist 230: 2404–2419. 33728642 10.1111/nph.17347

[nph71282-bib-0046] Liu M‐CJ , Yeh F‐LJ , Yvon R , Simpson K , Jordan S , Chambers J , Wu H‐M , Cheung AY . 2024. Extracellular pectin‐RALF phase separation mediates FERONIA global signaling function. Cell 187: 312–330. 38157854 10.1016/j.cell.2023.11.038

[nph71282-bib-0047] Liu S , Bartnikas LM , Volko SM , Ausubel FM , Tang D . 2016. Mutation of the glucosinolate biosynthesis enzyme cytochrome P450 83A1 monooxygenase increases camalexin accumulation and powdery mildew resistance. Frontiers in Plant Science 7: 227. 26973671 10.3389/fpls.2016.00227 PMC4774424

[nph71282-bib-0048] Liu Z , Chu X , Ren W , Cheng L , Liu C , Wang C , Gao S , Dai S , Li C . 2025. PCP‐B peptides and CrRLK1L receptor kinases control pollination via pH gating of aquaporins in Arabidopsis. Developmental Cell 60: 1336–1347. 39793583 10.1016/j.devcel.2024.12.026

[nph71282-bib-0049] Masachis S , Segorbe D , Turrà D , Leon‐Ruiz M , Fürst U , El Ghalid M , Leonard G , López‐Berges MS , Richards TA , Felix G *et al*. 2016. A fungal pathogen secretes plant alkalinizing peptides to increase infection. Nature Microbiology 1: 16043. 10.1038/nmicrobiol.2016.43 27572834

[nph71282-bib-0050] Mecchia MA , Santos‐Fernandez G , Duss NN , Somoza SC , Boisson‐Dernier A , Gagliardini V , Martínez‐Bernardini A , Fabrice TN , Ringli C , Muschietti JP *et al*. 2017. RALF4/19 peptides interact with LRX proteins to control pollen tube growth in Arabidopsis. Science 358: 1600–1603. 29242232 10.1126/science.aao5467

[nph71282-bib-0051] Merlot S , Leonhardt N , Fenzi F , Valon C , Costa M , Piette L , Vavasseur A , Genty B , Boivin K , Müller A *et al*. 2007. Constitutive activation of a plasma membrane H+ATPase prevents abscisic acid‐mediated stomatal closure. EMBO Journal 26: 3216–3226. 17557075 10.1038/sj.emboj.7601750 PMC1914098

[nph71282-bib-0052] Micali C , Göllner K , Humphry M , Consonni C , Panstruga R . 2008. The powdery mildew disease of Arabidopsis: a paradigm for the interaction between plants and biotrophic fungi. Arabidopsis Book 6: e0115. 22303240 10.1199/tab.0115 PMC3243333

[nph71282-bib-0053] Micali CO , Neumann U , Grunewald D , Panstruga R , O'Connell R . 2011. Biogenesis of a specialized plant–fungal interface during host cell internalization of *Golovinomyces orontii haustoria* . Cellular Microbiology 13: 210–226. 20880355 10.1111/j.1462-5822.2010.01530.x

[nph71282-bib-0054] Milne RJ , Dibley KE , Schnippenkoetter W , Mascher M , Lui ACW , Wang L , Lo C , Ashton AR , Ryan PR , Lagudah ES . 2019. The wheat Lr67 gene from the sugar transport protein 13 family confers multipathogen resistance in barley. Plant Physiology 179: 1285–1297. 30305371 10.1104/pp.18.00945 PMC6446772

[nph71282-bib-0055] Moussu S , Broyart C , Santos‐Fernandez G , Augustin S , Wehrle S , Grossniklaus U , Santiago J . 2020. Structural basis for recognition of RALF peptides by LRX proteins during pollen tube growth. Proceedings of the National Academy of Sciences, USA 117: 7494–7503. 10.1073/pnas.2000100117 PMC7132299 32165538

[nph71282-bib-0056] Moussu S , Lee HK , Haas KT , Broyart C , Rathgeb U , De Bellis D , Levasseur T , Schoenaers S , Fernandez GS , Grossniklaus U *et al*. 2023. Plant cell wall patterning and expansion mediated by protein‐peptide‐polysaccharide interaction. Science 382: 719–725. 37943924 10.1126/science.adi4720

[nph71282-bib-0057] Mravec J , Kračun SK , Rydahl MG , Westereng B , Pontiggia D , De Lorenzo G , Domozych DS , Willats WGT . 2017. An oligogalacturonide‐derived molecular probe demonstrates the dynamics of calcium‐mediated pectin complexation in cell walls of tip‐growing structures. The Plant Journal 91: 534–546. 28419587 10.1111/tpj.13574

[nph71282-bib-0058] Mucha S , Heinzlmeir S , Kriechbaumer V , Strickland B , Kirchhelle C , Choudhary M , Kowalski N , Eichmann R , Hückelhoven R , Grill E *et al*. 2019. The formation of a camalexin biosynthetic metabolon. Plant Cell 31: 2697–2710. 31511315 10.1105/tpc.19.00403 PMC6881122

[nph71282-bib-0059] Nawrath C , Métraux J‐P . 1999. Salicylic acid induction–deficient mutants of Arabidopsis express PR‐2 and PR‐5 and accumulate high levels of camalexin after pathogen inoculation. Plant Cell 11: 1393–1404. 10449575 10.1105/tpc.11.8.1393 PMC144293

[nph71282-bib-0060] Nekrasov V , Wang C , Win J , Lanz C , Weigel D , Kamoun S . 2017. Rapid generation of a transgene‐free powdery mildew resistant tomato by genome deletion. Scientific Reports 7: 482. 28352080 10.1038/s41598-017-00578-x PMC5428673

[nph71282-bib-0061] Ngou BPM , Jones JDG , Ding P . 2022. Plant immune networks. Trends in Plant Science 27: 255–273. 34548213 10.1016/j.tplants.2021.08.012

[nph71282-bib-0062] Noble JA , Bielski NV , Liu M‐CJ , DeFalco TA , Stegmann M , Nelson ADL , McNamara K , Sullivan B , Dinh KK , Khuu N *et al*. 2022. Evolutionary analysis of the LORELEI gene family in plants reveals regulatory subfunctionalization. Plant Physiology 190: 2539–2556. 36156105 10.1093/plphys/kiac444 PMC9706458

[nph71282-bib-0063] Pearce G , Yamaguchi Y , Munske G , Ryan CA . 2010. Structure–activity studies of RALF, Rapid Alkalinization Factor, reveal an essential – YISY – motif. Peptides 31: 1973–1977. 20800638 10.1016/j.peptides.2010.08.012

[nph71282-bib-0064] Peng Y , Yang J , Li X , Zhang Y . 2021. Salicylic acid: biosynthesis and signaling. Annual Review of Plant Biology 72: 761–791. 10.1146/annurev-arplant-081320-092855 33756096

[nph71282-bib-0065] Polonio Á , Pérez‐García A , Martínez‐Cruz J , Fernández‐Ortuño D , de Vicente A . 2021. The Haustorium of phytopathogenic fungi: a short overview of a specialized cell of obligate biotrophic plant parasites. In: Cánovas FM , Lüttge U , Risueño M‐C , Pretzsch H , eds. Progress in botany, vol. 82. Cham, Switzerland: Springer International Publishing, 337–355.

[nph71282-bib-0066] Raiola A , Lionetti V , Elmaghraby I , Immerzeel P , Mellerowicz EJ , Salvi G , Cervone F , Bellincampi D . 2011. Pectin methylesterase is induced in Arabidopsis upon infection and is necessary for a successful colonization by necrotrophic pathogens. Molecular Plant–Microbe Interactions 24: 432–440. 21171891 10.1094/MPMI-07-10-0157

[nph71282-bib-0067] Ried MK , Banhara A , Hwu F‐Y , Binder A , Gust AA , Höfle C , Hückelhoven R , Nürnberger T , Parniske M . 2019. A set of Arabidopsis genes involved in the accommodation of the downy mildew pathogen *Hyaloperonospora arabidopsidis* . PLoS Pathogens 15: e1007747. 31299058 10.1371/journal.ppat.1007747 PMC6625732

[nph71282-bib-0068] Rößling A‐K , Dünser K , Liu C , Lauw S , Rodriguez‐Franco M , Kalmbach L , Barbez E , Kleine‐Vehn J . 2024. Pectin methylesterase activity is required for RALF1 peptide signalling output. eLife 13: RP96943. 39360693 10.7554/eLife.96943 PMC11449480

[nph71282-bib-0069] Rzemieniewski J , Stegmann M . 2022. Regulation of pattern‐triggered immunity and growth by phytocytokines. Current Opinion in Plant Biology 68: 102230. 35588597 10.1016/j.pbi.2022.102230

[nph71282-bib-0070] Schade S , Leicher H , von Arx M , Monte I , Gronnier J , Stegmann M . 2025. The interplay of RALF structural and signaling functions in plant‐microbe interactions. PLoS Pathogens 21: e1013588. 41091714 10.1371/journal.ppat.1013588 PMC12527185

[nph71282-bib-0071] Scheinost L , Ludwig C , Höfflin N , Taskin AA , Marada A , Vögtle F‐N , Meisinger C , Köhn M . 2026. Synthetic trap‐peptides identify a TOM complex phosphatase – PP2A dephosphorylates Tom6. The FEBS Journal 293: 271–294. 40891445 10.1111/febs.70246 PMC12797000

[nph71282-bib-0072] van Schie CCN , Takken FLW . 2014. Susceptibility genes 101: how to be a good host. Annual Review of Phytopathology 52: 551–581. 10.1146/annurev-phyto-102313-045854 25001453

[nph71282-bib-0073] Schindelin J , Arganda‐Carreras I , Frise E , Kaynig V , Longair M , Pietzsch T , Preibisch S , Rueden C , Saalfeld S , Schmid B *et al*. 2012. Fiji: an open‐source platform for biological‐image analysis. Nature Methods 9: 676–682. 22743772 10.1038/nmeth.2019 PMC3855844

[nph71282-bib-0074] Schoenaers S , Lee HK , Gonneau M , Faucher E , Levasseur T , Akary E , Claeijs N , Moussu S , Broyart C , Balcerowicz D *et al*. 2024. Rapid alkalinization factor 22 has a structural and signalling role in root hair cell wall assembly. Nature Plants 10: 494–511. 38467800 10.1038/s41477-024-01637-8 PMC11494403

[nph71282-bib-0075] Sénéchal F , Habrylo O , Hocq L , Domon J‐M , Marcelo P , Lefebvre V , Pelloux J , Mercadante D . 2017. Structural and dynamical characterization of the pH‐dependence of the pectin methylesterase‐pectin methylesterase inhibitor complex. Journal of Biological Chemistry 292: 21538–21547. 29109147 10.1074/jbc.RA117.000197 PMC5766959

[nph71282-bib-0076] Shen Q , Bourdais G , Pan H , Robatzek S , Tang D . 2017. *Arabidopsis* glycosylphosphatidylinositol‐anchored protein LLG1 associates with and modulates FLS2 to regulate innate immunity. Proceedings of the National Academy of Sciences, USA 114: 5749–5754. 10.1073/pnas.1614468114 PMC5465910 28507137

[nph71282-bib-0077] Stegmann M , Monaghan J , Smakowska‐Luzan E , Rovenich H , Lehner A , Holton N , Belkhadir Y , Zipfel C . 2017. The receptor kinase FER is a RALF‐regulated scaffold controlling plant immune signaling. Science 355: 287–289. 28104890 10.1126/science.aal2541

[nph71282-bib-0078] Sun M , Lu B , Yang Y , Fan J , Ren W , Chu X , Gao Y , Wu J , Wang J , Ke H *et al*. 2025. Unveiling FERONIA receptor kinase–mediated cellular mechanisms with a small‐molecule inhibitor. Proceedings of the National Academy of Sciences, USA 122: e2515322122. 10.1073/pnas.2515322122 PMC12625892 41196348

[nph71282-bib-0079] Tang J , Wu D , Li X , Wang L , Xu L , Zhang Y , Xu F , Liu H , Xie Q , Dai S *et al*. 2022. Plant immunity suppression via PHR1‐RALF‐FERONIA shapes the root microbiome to alleviate phosphate starvation. EMBO Journal 41: e109102. 35146778 10.15252/embj.2021109102 PMC8922250

[nph71282-bib-0080] Tang W , Lin W , Zhou X , Guo J , Dang X , Li B , Lin D , Yang Z . 2022. Mechano‐transduction via the pectin‐FERONIA complex activates ROP6 GTPase signaling in *Arabidopsis* pavement cell morphogenesis. Current Biology 32: 508–517. 34875231 10.1016/j.cub.2021.11.031

[nph71282-bib-0081] Thynne E , Saur IML , Simbaqueba J , Ogilvie HA , Gonzalez‐Cendales Y , Mead O , Taranto A , Catanzariti A‐M , McDonald MC , Schwessinger B *et al*. 2017. Fungal phytopathogens encode functional homologues of plant rapid alkalinization factor (RALF) peptides. Molecular Plant Pathology 18: 811–824. 27291634 10.1111/mpp.12444 PMC6638259

[nph71282-bib-0082] Vogel JP , Raab TK , Schiff C , Somerville SC . 2002. PMR6, a pectate lyase–like gene required for powdery mildew susceptibility in Arabidopsis. Plant Cell 14: 2095–2106. 12215508 10.1105/tpc.003509 PMC150758

[nph71282-bib-0083] Vogel JP , Raab TK , Somerville CR , Somerville SC . 2004. Mutations in PMR5 result in powdery mildew resistance and altered cell wall composition. The Plant Journal 40: 968–978. 15584961 10.1111/j.1365-313X.2004.02264.x

[nph71282-bib-0084] Wang H , Li X , Zhai K , Rhodes J , Sang T , Zhao J , Gao Y , Ma S , Song B , Pan Q *et al*. 2026. A regulatory network promotes apoplastic alkalinization to prime plant immunity in tissues distal to site of infection. Cell 189: 1389–1406. 41742413 10.1016/j.cell.2026.01.027

[nph71282-bib-0085] Weis C , Pfeilmeier S , Glawischnig E , Isono E , Pachl F , Hahne H , Kuster B , Eichmann R , Hückelhoven R . 2013. Co‐immunoprecipitation‐based identification of putative BAX INHIBITOR‐1‐interacting proteins involved in cell death regulation and plant–powdery mildew interactions. Molecular Plant Pathology 14: 791–802. 23782494 10.1111/mpp.12050 PMC6638788

[nph71282-bib-0086] Willats WGT , Orfila C , Limberg G , Buchholt HC , van Alebeek G‐JWM , Voragen AG , Marcus SE , Christensen TMIE , Mikkelsen JD , Murray BS *et al*. 2001. Modulation of the degree and pattern of methyl‐esterification of pectic homogalacturonan in plant cell walls: implications for pectin methyl esterase action, matrix properties, and cell adhesion. Journal of Biological Chemistry 276: 19404–19413. 11278866 10.1074/jbc.M011242200

[nph71282-bib-0087] Xiao Y , Stegmann M , Han Z , DeFalco TA , Parys K , Xu L , Belkhadir Y , Zipfel C , Chai J . 2019. Mechanisms of RALF peptide perception by a heterotypic receptor complex. Nature 572: 270–274. 31291642 10.1038/s41586-019-1409-7

[nph71282-bib-0088] Xu F , Gonneau M , Faucher E , Habrylo O , Lefebvre V , Domon J‐M , Martin M , Sénéchal F , Peaucelle A , Pelloux J *et al*. 2022. Biochemical characterization of pectin methylesterase inhibitor 3 from *Arabidopsis thaliana* . The Cell Surface 8: 100080. 36147700 10.1016/j.tcsw.2022.100080 PMC9486134

[nph71282-bib-0089] Yamada K , Saijo Y , Nakagami H , Takano Y . 2016. Regulation of sugar transporter activity for antibacterial defense in Arabidopsis. Science 354: 1427–1430. 27884939 10.1126/science.aah5692

[nph71282-bib-0090] Yang T , Wang L , Li C , Liu Y , Zhu S , Qi Y , Liu X , Lin Q , Luan S , Yu F . 2015. Receptor protein kinase FERONIA controls leaf starch accumulation by interacting with glyceraldehyde‐3‐phosphate dehydrogenase. Biochemical and Biophysical Research Communications 465: 77–82. 26232644 10.1016/j.bbrc.2015.07.132

[nph71282-bib-0091] Yeats TH , Sorek H , Wemmer DE , Somerville CR . 2016. Cellulose deficiency is enhanced on hyper accumulation of sucrose by a H+‐coupled sucrose symporter. Plant Physiology 171: 110–124. 27013021 10.1104/pp.16.00302 PMC4854719

[nph71282-bib-0092] Zecua‐Ramirez P , Jha V , Stegmann M . 2026. Peptide signaling at the intersection of growth, nutrient sensing, and stress responses. The Plant Journal 125: e70733. 41693079 10.1111/tpj.70733 PMC12907539

[nph71282-bib-0093] Zhai K , Derbyshire P , Zhang S , Choi S , Wang L , Song B , Kinoshita T , Zhou J‐M , Menke FLH , Bender KW *et al*. 2026. A phosphorelay circuit drives extracellular alkalinization in receptor kinase‐mediated immune and cell‐wall damage signaling. Molecular Cell 86: 1797–1814. 42019498 10.1016/j.molcel.2026.03.035

[nph71282-bib-0094] Zhang K , Halitschke R , Yin C , Liu C‐J , Gan S‐S . 2013. Salicylic acid 3‐hydroxylase regulates *Arabidopsis* leaf longevity by mediating salicylic acid catabolism. Proceedings of the National Academy of Sciences, USA 110: 14807–14812. 10.1073/pnas.1302702110 PMC3767541 23959884

[nph71282-bib-0095] Zhang X , Peng H , Zhu S , Xing J , Li X , Zhu Z , Zheng J , Wang L , Wang B , Chen J *et al*. 2020. Nematode‐encoded RALF peptide mimics facilitate parasitism of plants through the FERONIA receptor kinase. Molecular Plant 13: 1434–1454. 32896643 10.1016/j.molp.2020.08.014

[nph71282-bib-0096] Zhang Y , Zhao L , Zhao J , Li Y , Wang J , Guo R , Gan S , Liu C‐J , Zhang K . 2017. S5H/DMR6 encodes a salicylic acid 5‐hydroxylase that fine‐tunes salicylic acid homeostasis. Plant Physiology 175: 1082–1093. 28899963 10.1104/pp.17.00695 PMC5664462

[nph71282-bib-0097] Zhao C , Zayed O , Yu Z , Jiang W , Zhu P , Hsu C‐C , Zhang L , Tao WA , Lozano‐Durán R , Zhu J‐K . 2018. Leucine‐rich repeat extensin proteins regulate plant salt tolerance in *Arabidopsis* . Proceedings of the National Academy of Sciences, USA 115: 13123–13128. 10.1073/pnas.1816991115 PMC6305001 30514814

[nph71282-bib-0098] Zhong S , Li L , Wang Z , Ge Z , Li Q , Bleckmann A , Wang J , Song Z , Shi Y , Liu T *et al*. 2022. RALF peptide signaling controls the polytubey block in Arabidopsis. Science 375: 290–296. 35050671 10.1126/science.abl4683 PMC9040003

[nph71282-bib-0099] Zhou F , Andersen CH , Burhenne K , Fischer PH , Collinge DB , Thordal‐Christensen H . 2000. Proton extrusion is an essential signalling component in the HR of epidermal single cells in the barley‐powdery mildew interaction. The Plant Journal 23: 245–254. 10929118 10.1046/j.1365-313x.2000.00777.x

[nph71282-bib-0100] Zhu S , Fu Q , Xu F , Zheng H , Yu F . 2021. New paradigms in cell adaptation: decades of discoveries on the CrRLK1L receptor kinase signalling network. New Phytologist 232: 1168–1183. 34424552 10.1111/nph.17683

[nph71282-bib-0101] Zimmerli L , Stein M , Lipka V , Schulze‐Lefert P , Somerville S . 2004. Host and non‐host pathogens elicit different jasmonate/ethylene responses in Arabidopsis. The Plant Journal 40: 633–646. 15546348 10.1111/j.1365-313X.2004.02236.x

