# Supplementary material for: Endogenous RALF peptide function is required for powdery mildew host colonization
Source: New Phytol. 2026 May 27;251(4):2073–90. doi: 10.1111/nph.71282 (PMC13373830; doi:10.1111/nph.71282)
Supplement: Supplementary file 1 — Fig. S1 Quantification of fungal growth on mutants of RALF binding proteins. Fig. S2 Images of plant lines used in this study. Fig. S3 Characterization of CRISPR ralf mutants. Fig. S4 Regulation of RALF peptides during powdery mildew infection. Fig. S5 FER‐ and RALF‐mediated powdery mildew susceptibility is most likely not directly linked to MLO. Fig. S6 Characterization of fer‐4 CRISPR myc2. Fig. S7 FER interactors detected by Co‐IP‐MS. Fig. S8 Powdery mildew dependent FER interactors detected by Co‐IP‐MS. Fig. S9 Role of apoplastic pH and PMEs for Ecr infection success. Fig. S10 Analysis of FER‐independent RALF23 functions during Ecr infection. Fig. S11 Cell wall localization of mCherry‐RALF23 peptide variants. Table S1 Primers used in this study. Table S2 CRISPR target sites. Table S3 Plant lines used in this study. Table S4 Identification of RALF‐like peptides from phytopathogenic fungi species reported to be able to infect Arabidopsis. Please note: Wiley is not responsible for the content or functionality of any Supporting Information supplied by the authors. Any queries (other than missing material) should be directed to the New Phytologist Central Office. [file NPH-251-2073-s001.docx]

## *New Phytologist* Supporting Information

Article title: Endogenous RALF peptide function is required for powdery mildew host colonization

Authors: Henriette Leicher, Sebastian Schade, Jan W. Huebbers, Kristina S. Munzert-Eberlein, Genc Haljiti, David Biermann, Athanasios Makris, Xiaoxuan Zhu, Yashank Chauhan, Christina Ludwig, Marion C. Müller, Toshinori Kinoshita, Timo Engelsdorf, Julien Gronnier, Martina K. Ried-Lasi, Aurélien Boisson-Dernier, Ralph Hückelhoven and Martin Stegmann

Article acceptance date: 30 April 2026

The following Supporting Information is available for this article:

**Figure S1: Quantification of fungal growth on mutants of RALF binding proteins.** A-C) Amount of fungal DNA normalized to plant DNA (5 dpi) upon *Ecr* infection of the indicated genotypes. Data points indicate independent biological replicates. A) n=14, B) n=7–9, C) n=3. C) Data points with different symbols indicate independent biological replicates. All experiments were done at least three times with similar results. Dpi: Days post infection, FER: FERONIA, LLG1: LORELEI-LIKE GPI ANCHORED PROTEIN 1, LRX: LEUCINE-RICH REPEAT EXTENSIN.

**Figure S2: Images of plant lines used in this study.** Representative images of 5-week-old plants used in this study. FER: FERONIA, RALF: RAPID ALKALINIZATION FACTOR, LLG1: LORELEI-LIKE GPI ANCHORED PROTEIN 1, LRX: LEUCINE-RICH REPEAT EXTENSIN, OST2: OPEN STOMATA 2, LRR: LEUCIN-RICH REPEAT, GFP: GREEN FLUORESCENT PROTEIN, PMEI: PECTIN METHYL ESTERASE INHIBITOR, OE: Overexpression

**Figure S3: Characterization of CRISPR *ralf* mutants.** A) Expression levels of the indicated *RALF* genes in the vegetative rosette. Data was obtained using ePlant browser (https://bar.utoronto.ca/eplant/) and is based on (Schmid *et al.*, 2005). B) Characterization of CRISPR *ralf3x*. Schematic diagram of *RALF22, RALF23* and *RALF33* gene structure and the CRISPR-Cas9-mediated mutation pattern detected by DNA sequencing. The mature RALF peptide is indicated in blue. C) Characterization of CRISPR *ralf7x*. Schematic diagram of *RALF1, RALF18, RALF22, RALF23*, *RALF31,* *RALF33* and *RALF34* gene structure and the CRISPR-Cas9-mediated mutation pattern detected by DNA sequencing. The mature RALF peptide is indicated in blue. D) Quantification of the petiole length of 4-week-old Col-0, *fer-4*, CRISPR *ralf3x* and CRISPR *ralf7x* plants. Each data point represents the average length of petioles on a fully grown rosette. Mean ± SD, n=7 pooled from two independent experiments (Tukey’s multiple comparisons test, a-b p<0.0001). E) Amount of fungal DNA normalized to plant DNA (5 dpi) upon *Ecr* infection of the indicated genotypes. Data points with different symbols indicate independent biological replicates n=3-5, F-G) RT-qPCR of *RALF23* in adult leaves of the indicated plant lines. Housekeeping gene *UBQ5,* n=2. Data points with different symbols indicate independent biological replicates. D-E) were performed at least two times with identical results. F–G) were performed twice with identical results. Dpi: Days post infection, SD: standard deviation, bp: base pairs, FER: FERONIA, RALF: RAPID ALKALINIZATION FACTOR, UBQ5: UBIQUITIN 5.

**Figure S4: Regulation of RALF peptides during powdery mildew infection.** RT-qPCR of the indicated *RALF* genes in Col-0 (blue) and *fer-4* (red) at 0 dpi (circle), 1 dpi (square), 4 dpi (triangle), 5 dpi (diamond). Housekeeping gene *UBQ5*. Mean ± SD, n=4-5, data points indicate independent biological replicates. (Dunn’s multiple comparison test against 0 dpi within the respective genotype, p- values < 0.05 are indicated in the graph, all other comparisons were not significant. Dpi: Days post infection, SD: standard deviation, FER: FERONIA, RALF: RAPID ALKALINIZATION FACTOR, UBQ5: UBIQUITIN 5

**Figure S5: FER- and RALF-mediated powdery mildew susceptibility is most likely not directly linked to MLO.** A) Confocal microscopy images of fungal spores penetrating p35S::MLO2-mCherry and pFER::FER-GFP lines (2 dpi). Dotted lines indicate fungal spores, white arrow marks the penetration site. Scale bar represents 50 µm. B) Quantification of hyphal branching (2 dpi). Mean ± SD, n=77–98 pooled from two independent experiments (Mann Whitney test, ns: not significant). C) Penetration efficiency of *Ecr* on the indicated genotypes (calculated as the percentage of successful penetrations from all counted interactions, 2 dpi). Mean ± SD, n=2–4. D) Confocal microscopy images of fungal spores at 1 dpi. Callose depositions were stained using methyl blue (turquoise). Fungal structures were stained with propidium iodide (red). Scale bar represents 50 µm. E) Quantification of methyl blue fluorescence around the fungal penetration site, indicating the amount of callose deposition. Mean ± SD, n=43-45 pooled from three independent experiments (Dunn’s multiple comparisons test). F) Conidiophores per field of view (5 dpi) of fungal colonies grown on Col-0 plants. Spores from the indicated genotypes were used for infection. Mean ± SD, n=218-336 pooled from three independent experiments (Dunn’s multiple comparisons test). G) Quantification of trichome numbers per leaf area on the indicated genotypes. Mean ± SD, n=27 pooled from three independent experiments (Dunn’s multiple comparisons test, a-b p<0.0001). H) Microscopic images of aniline blue-stained trichomes of Col-0, *fer-4* and CRISPR *ralf7x*. Scale bar in the upper row represents 50 µm, scale bar in the lower panel represents 500 µm. I) Quantification of trichome branching on the indicated genotypes. Mean ± SD, n=27 pooled from three independent experiments (Dunn’s multiple comparisons test, statistical analysis was performed on each category individually, 2 branches: a-b p=0.0002; 3 branches: a-b p=0.0259; a-c, b-c p<0.0001; 4 branches: a-b p<0.0001). All experiments were performed three times with similar results, except B), which was performed twice with identical results. Dpi: Days post infection, SD: standard deviation, *Ecr:* *Erysiphe cruciferarum*, FER: FERONIA, RALF: RAPID ALKALINIZATION FACTOR, LLG1: LORELEI-LIKE GPI ANCHORED PROTEIN 1, GFP: GREEN FLUORESCENT PROTEIN, MLO2: MILDEW LOCUS O 2, BF: Bright field.

**Figure S6: Characterization of *fer-4* CRISPR *myc2.*** A) Quantification of glycosylated (green) 2,3-DHBA in untreated and *Ecr* infected (5 dpi) leaves of the indicated genotypes. Mean ± SD, n=3 pooled from three independent experiments (Tukey’s multiple comparisons test, glycosylated: a-b p≤0.0324). B) Fungal spores/mg fresh weight (FW) of *Hyaloperonospora arabidopsidis* (*Hpa*) grown on the indicted genotypes. Mean ± SD, n=19-23 pooled from three independent experiments (Dunn’s multiple comparison test, a-b p≤0.0129). All experiments were repeated at least three times with similar results. C) Characterization of *fer-4* CRISPR *myc2*. Schematic diagram of *MYC2* gene structure and the CRISPR-Cas9-mediated mutation pattern detected by DNA sequencing. D) RT-qPCR of *MYC2* in untreated (grey) adult leaves and upon infection with *Ecr* (5 dpi, green). Housekeeping gene *UBQ5*. Mean ± SD, n=3, data points with different symbols indicate independent biological replicates (Tukey’s multiple comparisons test, a-b p≤0.0358). E) Images of 4-week-old Col-0, *fer-4* and *fer-4* CRISPR *myc2* plants. F) Amount of fungal DNA normalized to plant DNA (5 dpi) upon *Ecr* infection of the indicated genotypes, n=3. Data points with different symbols indicate independent biological replicates. A, B, D and F were performed three times with similar results. Dpi: Days post infection, SD: standard deviation, *Ecr:* *Erysiphe cruciferarum*, FER: FERONIA, LRX: LEUCINE-RICH REPEAT EXTENSIN, 2,3-DHBA: 2-3 Dihydroxybenzoic acid, UBQ5: UBIQUITIN 5, FW: Fresh weight.

**Figure S7: FER interactors detected by CoIP-MS.** A) Volcano plot of detected FER-GFP interactors. Significantly enriched proteins (FDR=0.05 and fold change (FER-GFP/Lti6b-GFP) >2) are marked with colored circles according to the legend. Non-significant proteins are colored grey. B) List of significant FER-GFP interactors compared to Lti6b-GFP control. Data was obtained from biological triplicates for FER-GFP and biological quadruplets for Lti6b-GFP. FDR: False discovery rate.

**Figure S8: Powdery mildew dependent FER interactors detected by CoIP-MS.** A) Volcano plot of detected FER-GFP interactors after *Ecr* infection (1 dpi). Significantly enriched proteins (FDR=0.05 and fold change (*Ecr* infection (1dpi)/Untreated) >2) are marked in black. Non-significant proteins are colored grey. B) List of significant FER-GFP interactors at 1 dpi with *Ecr*, compared to the untreated FER-GFP control. Data was obtained from biological triplicates for each treatment. FDR: False discovery rate.

**Figure S9: Role of apoplastic pH and PMEs for *Ecr* infection success.** A) Quantification of the band intensities detected in western blot probed with α-AHA and α-Thr^947P^ antibodies. Band intensities were measured using Image J software. Data points indicate individual experiments. Data points from the same experiment are marked with the same color. B) AHA phosphorylation upon infection with *Ecr* (3 dpi). Western blots were probed with α-AHA and α-Thr^947P^ antibodies. C) Conidiophores per field of view (5 dpi) after pretreatment with H_2_O (blue), MgCl_2_ 10 mM pH 7 (grey), HEPES buffer 10 mM pH 7.5 (red) before *Ecr* infection. Mean ± SD, n=53-153 pooled from three independent experiments (Dunn’s multiple comparison, a-b, a-dc, a-bc, a-cd, a-d, b-d p<0.0001, b-cd p=0.0245). D) Confocal images of fungal structures stained with COS-488. Scale bar represents 50 µm E) Conidiophores per field of view (5 dpi) of fungal colonies grown upon *Ecr* infection of the indicated genotypes. Mean ± SD, n=161-338 pooled from three independent experiments (Dunn’s multiple comparisons test, a-b p<0.0001). All experiments were repeated at least three times with similar results. Dpi: Days post infection, SD: standard deviation, FER: FERONIA, AHA: AUTOINHIBITED H^+^-ATPase, PME: PECTIN METHYL ESTERASE, CBB: Coomassie brilliant blue, *Ecr: Erysiphe cruciferarum,* Fluo. Int.: Fluorescence intensity.

**Figure S10: Analysis of FER-independent RALF23 functions during *Ecr* infection.** A) Characterization of pUBQ10::SYP122-pHusion CRISPR *fer-4*. Schematic diagram of *FERONIA* gene structure and the CRISPR-Cas9-mediated mutation pattern detected by DNA sequencing. B) Conidiophores per field of view (5 dpi) after pretreatment with H_2_O (blue), 100 nM RALF23 (turquoise), or 10 nM RALF23 (green). Mean ± SD, n=210-259 pooled from four independent experiments (Dunn’s multiple comparisons test, genotypes were analyzed separately. C) Quantification of the EGFP/mRFP1 3 h post infiltration of 1 µM RALF23 in H_2_O or MES pH 5.5. Mean ± SD, n=21-24 pooled from three independent experiments (Dunn’s multiple comparisons test a-b≤0.009, Mann Whitney test, **p=0.0017, ns=not significant). D) PME activity normalized to Col-0. Mean ± SD, n=4-5, data points with different symbols indicate independent biological replicates (Tukey’s multiple comparisons test, not significant). (E) RT-qPCR of *RALF23* in untreated adult leaves of the indicated plant lines. Housekeeping gene *UBQ5*. n=3, data points with different symbols indicate independent biological replicates. B-E) All experiments were performed at least three times with similar results. Dpi: Days post infection, SD: standard deviation, FER: FERONIA, RALF: RAPID ALKALINIZATION FACTOR, EGFP: enhanced GREEN FLUORESCENCE PROTEIN, RFP: RED FLUORESCENCE PROTEIN, PME: PECTIN METHYL ESTERASE.

**Figure S11: Cell wall localization of mCherry-RALF23 peptide variants.** A) Representative z-stack images of epidermal cell expressing different variants of pR23::mCherry-RALF23. Panel 1: mCherry-RALF23 localization in untreated epidermal cells, panel 2-4: Epidermal cells after mannitol treatment (0.5 M), panel 3 and 4 show the image section marked in panel 2. Scale bar represents 50 µm. cw: Cell wall, pm: Plasma membrane. RALF: RAPID ALKALINIZATION FACTOR, WT: Wild type.

**Table S1: Primers used in this study**

| **Primers used for cloning** | | | |
| --- | --- | --- | --- |
| **Name** | | **Sequence (5' - 3')** | **Purpose** |
| RALF23_Esp3I_F | | ttCGTCTCaaatgagaggactctccagaaac | p35S::RALF23 |
| RALF23_Esp3I_R | | ttCGTCTCaactatgagcgccggcagcgag |  |
| R23^Y113AY114A^_F | | ttCGTCTCctgccgccaattgtcgacgtg | p35S::RALF23^Y113A/Y1114A^ |
| R23^Y113AY114A^_R | | ttCGTCTCcggcagatgcgccgcgacgtg |  |
| R23^Y93AY96A^_F | | ttCGTCTCatcagcgctggtgcgctgaggag | p35S::RALF23^Y93A/Y96A^ |
| R23^Y93AY96A^_R | | ttCGTCTCgctgatgtacctcctcgtagc |  |
| MLO2_Esp3I_F | | ttCGTCTCaaatggcagatcaagtaaaag | p35S::MLO2-mCherry |
| MLO2_Blunt_R | | tttcttaaaagaaaaatctcttaa (5'Phosphorylated) |  |
| pR23_BpiI_F | | ttGAAGACaacgagtggagaacctacgatttc | pR23::mCherryRALF23 |
| pR23_BpiI_R | | ttGAAGACaacatttttcttctgtacactgtag |  |
| **Primers used for RT-qPCR** | | | |
| **Name** | **Sequence (5' - 3')** | | **Purpose** |
| Ath_Rubisco_F | TGATGGACGGTACTGGACAA | | Quantification of plant DNA |
| Ath_Rubsico_R | GAAGCTTGGTGGCTTGTAGG | |  |
| Ecr_Tubulin_F | TGACAGCCCGGAATGAGT | | Quantification of fungal DNA |
| Ecr_Tubulin_R | TTGTCTTCGTTTCCCAGGTC | |  |
| RALF23_qPCR_F | TTCATAACTGGTCCGTCGCC | | RALF23 |
| RALF23_qPCR_R | AGCTAATATGCGCCGGTTGA | |  |
| RALF1_qPCR_F | CATCTCTTCACCTCCGGTCC | | RALF1 |
| RALF1_qPCR_R | CTATTGAACCGTGGCAGCCT | |  |
| RALF18_qPCR_F | GTCGGATCTCGTCCGGTAAA | | RALF18 |
| RALF18_qPCR_R | CGGTTTACCATCTGGTTTGGC | |  |
| RALF22_qPCR_F | GCGGAAGAAGAAGAGATGGAG | | RALF22 |
| RALF22_qPCR_R | TTGTAGTACGACGCACCAC | |  |
| RALF31_qPCR_F | ACGGAGAGATCGACGCAATG | | RALF31 |
| RALF31_qPCR_R | CCATCAGTACTCTCCGGCTG | |  |
| RALF33_qPCR_F | CTCACCGTCCACTTCTTATTC | | RALF33 |
| RALF33_qPCR_R | CGTTGTTGCTAAAATACGCC | |  |
| RALF34_qPCR_F | CTCCTCCTCATTCTCTCTCTTC | | RALF34 |
| RALF34_qPCR_R | TCGTCTTCCTCCGTAACTTC | |  |
| UBQ5_qPCR_F | ACTCCTTCCTCAAACGCTGA | | UBQ5 |
| UBQ5_qPCR_R | CCAAGCCGAAGAAGATCAAG | |  |
| MYC2_qPCR_F | GTGCGGGATTAGCTGGTAAA | | MYC2 |
| MYC2_qPCR_R | ATGCATCCCAAACACTCCTC | |  |
| PR1_qPCR_F | CGGAGCTACGCAGAACAACT | | PR1 |
| PR1_qPCR_R | CAGACAAGTCACCGCTACCC | |  |
| JAZ10_qPCR_F | GAGAAGCGCAAGGAGAGATTAG | | JAZ10 |
| JAZ10_qPCR_R | CTTAGTAGGTAACGTAATCTCC | |  |
| VSP2_qPCR_F | CGTCGATTCGAAAACCATCT | | VSP2 |
| VSP2_qPCR_R | GGCACCGTGTCGAAGTCTAT | |  |
| **Primers used for genotyping** | | | |
| **Name** | **Sequence (5' - 3')** | | **Purpose** |
| GG pUC18 Seq-F | GTATCACGAGGCCCTTTCGT | | Inserts in GoldenGate pUC18 |
| GG pUC18 Seq-R | TAATGAATCGGCCAACGC | |  |
| pGGPlant-XL-AB-insert_Fwd | aaacgacaatctgagctccacc | | Insert in GoldenGate pGGPlantXL-AB |
| pGGPlantXL-AB-insert_Rev | cggtgtcatctatgttactagat | |  |
| RALF1_F | CAAAATTAAAAAATCGAGTCCAC | | CRISPR RALF1 |
| RALF1_R | GCTTGATGGATCAAAGAAAAC | |  |
| RALF14_F | GCTCTTAATCTTCGCCG | | CRISPR RALF14 |
| RALF14_R | GTAAACCGGTAACAATGTG | |  |
| RALF18_F | CATCACAAACCATTTCAACG | | CRISPR RALF18 |
| RALF18_R | CACACCAACGAAAGGAAG | |  |
| RALF22_F | GTTTCCAAGAGAGAAAGAGAG | | CRISPR RALF22 |
| RALF22_R | GAAAATCGAGAAGAGAGACAG | |  |
| RALF23_F | CTTCATAGTTCGTGCACAG | | CRISPR RALF23 |
| RALF23_R | CTAATAATCCGCAATGAACTTAG | |  |
| RALF31_F | CTTCTTCTGCTTAGAGACAG | | CRISPR RALF31 |
| RALF31_R | CAGCAATGCAAAAGCTTAATC | |  |
| RALF33_F | CCTCAGAGAAAGAAGAAGAAAG | | CRISPR RALF33 |
| RALF33_R | GCAATGCAAAGTATAAACATAG | |  |
| RALF34_F | CCTCTTTACATGTGACTTCC | | CRISPR RALF34 |
| RALF34_R | CACGGTTTACAAACAAACATC | |  |
| MYC2_F | GGAACACACGAAGGTTGGAC | | CRISPR MYC2 |
| MYC2_R | CTGCTACTTCTTTCACGACGG | |  |
| FER_F | CGTAACCAAACGAACATG | | CRISPR FER |
| FER_R | GGTAAGTGAAACCAGAGTC | |  |

**Table S2: CRISPR target sites**

| **Target site** | **Sequence** | **Purpose** |
| --- | --- | --- |
| RALF1.1 | ACACTCTGCTATTGAACCG | Generation of CRISPR *ralf* mutants |
| RALF1.2 | AATTGTCAGAACGGAGCTC |  |
| RALF14.1 | GATGCATTTTCCACTCAAC |  |
| RALF18.1 | GTGGTTGCAGTGCCGCTAC |  |
| RALF22.1 | GTTCGATTCAGACATAAGC |  |
| RALF22.2 | CGTACTACAACTGCCAGCG |  |
| RALF23.1 | CCTCGTAGCTAATATGCGC |  |
| RALF23.2 | CAGCCACGAGAGTAAGGAT |  |
| RALF31.1 | TGGCGCAGAAACGGTACAT |  |
| RALF31.2 | TTTGTGTCTCTAGCGCATC |  |
| RALF33.1 | CGATCGAGTCGAAATGCAA |  |
| RALF33.2 | TAGCGCTATTACTCGTTGC |  |
| RALF34.1 | CTCTCACTCTTCTACCCGA |  |
| RALF34.2 | CCGTTCATCCGTATAGCCG |  |
| MYC2.1 | CCGCCGGAGTAGAAAACGG | Generation of *fer*-4 CRISPR *myc2* |
| FER.1 | GACGATTAAGTATCCTACG | Generation of pUBQ10::SYP122-apopHusion CRISPR *fer*-4 |
| FER.2 | TGATCGATGAAGATCACAG |  |

**Table S3: Arabidopsis plant lines used in this study.**

| Plant line | Genetic Background | Reference |
| --- | --- | --- |
| *fer-4* | Col-0 | (Duan *et al.*, 2010) |
| *fer-2* | Col-0 | (Deslauriers & Larsen, 2010) |
| pFER::FER-GFP | *fer-4* | (Duan *et al.*, 2010) |
| *llg1-2* | Col-0 | (Li *et al.*, 2015) |
| *llg1-3* | Col-0 | (Shen *et al.*, 2017) |
| *lrx1/2/3* | Col-0 | (Draeger *et al.*, 2015) |
| *lrx1/2/3/4/5* | Col-0 | (Herger *et al.*, 2020) |
| p35S::LRR4-citrine | Col-0 | (Fabrice *et al.*, 2018) |
| *ralf1/22/23/33* | Col-0 | (Lan *et al.*, 2023) |
| CRISPR *ralf3x* | Col-0 | Generated in this study |
| CRISPR *ralf7x* | Col-0 | Generated in this study |
| p35S::RALF23 | CRISPR *ralf7x* | Generated in this study |
| p35S::RALF23^Y93A/Y96A^ | CRISPR *ralf7x* | Generated in this study |
| p35S::RALF23^Y113A/Y114A^ | CRISPR *ralf7x* | Generated in this study |
| pR23::mCherry-RALF23 | CRISPR *ralf7x* | Generated in this study |
| pR23::mCherry-RALF23^Y93A/Y96A^ | CRISPR *ralf7x* | Generated in this study |
| pR23::mCherry-RALF23^Y113A/Y114A^ | CRISPR *ralf7x* | Generated in this study |
| *mlo2/6/12* | Col-0 | (Consonni *et al.*, 2006) |
| CRISPR *myc2* | *fer-4* | Generated in this study |
| PMEI3-OE | Col-0 | (Peaucelle *et al.*, 2008) |
| *ost2-2D* | Col-0 | (Merlot *et al.*, 2007) |
| *pme3* | Col-0 | (Guénin *et al.*, 2011) |
| *pme17* | Col-0 | (Del Corpo *et al.*, 2020) |
| pUBQ10::SYP122-pHusion | Col-0 | (Kesten *et al.*, 2019) |
| pUBQ10::SYP122-pHusion/CRISPR *fer* | pUBQ10::SYP122-pHusion | Generated in this study |
| pFER:FER-GFP | *fer-4* | (Chakravorty *et al.*, 2018) |
| pFER:FER^K565R^-GFP | *fer-4* | (Chakravorty *et al.*, 2018) |
| p35S::RALF23-GFP | Col-0 | (Dobón *et al.*, 2015) |
| *fer-2* x p35S::RALF23-GFP | *fer-2* | (Stegmann *et al.*, 2017) |
| pFER::FER^WT^-GFP | *fer-4* | (Xiao *et al.*, 2019) |
| pFER::FER^N303Y^-GFP | *fer-4* | (Xiao *et al.*, 2019) |
| pFER::FER^G257A^-GFP | *fer-4* | (Xiao *et al.*, 2019) |
| p35S::Lti6b-GFP | Col-0 | (Kadota *et al.*, 2014) |

**Table S4: Identification of RALF-like peptides from phytopathogenic fungi species reported to be able to infect Arabidopsis.**

| Species | Significant hits in predicted proteins (E-values < 10e^-05)^ | Significant hits in translated genome assembly (E-values < 10e^-05)^ | GenBank assembly  accession number | Reference |
| --- | --- | --- | --- | --- |
| Fusarium oxysporum NRRL 32931 | 1 | 2 | GCA_000271745.2 | (DeIulio *et al.*, 2018) |
| Pyrenophora tritici-repentis Pt-1C-BFP | 1 | 1 | GCA_000149985.1 | (Manning *et al.*, 2013) |
| Golovinomyces cichoracearum^a^ UMSG1 | 0 | 0 | GCA_003611235.1 | (Wu *et al.*, 2018) |
| Erysiphe neolycopersicum^a^ UMSG2 | 0 | 0 | GCA_003610855 | (Wu *et al.*, 2018) |
| Erysiphe necator EnFRAME01 | 0 | 0 | GCA_024703715.1 | (Zaccaron *et al.*, 2023) |
| *Blumeria graminis* f.sp. *tritici* CHE_96224 | 0 | 0 | GCA_900519115.1 | (Müller *et al.*, 2019) |
| *Podosphera xanthii* YZU573 | 0 | 0 | GCA_028751805.1 | (Xu & Chen, 2023) |

**References**

**Chakravorty D, Yu Y, Assmann SM. 2018.** A kinase-dead version of FERONIA receptor-like kinase has dose-dependent impacts on rosette morphology and RALF1-mediated stomatal movements. *FEBS Letters* **592**: 3429-3437.

**Consonni C, Humphry ME, Hartmann HA, Livaja M, Durner J, Westphal L, Vogel J, Lipka V, Kemmerling B, Schulze-Lefert P, et al. 2006.** Conserved requirement for a plant host cell protein in powdery mildew pathogenesis. *Nat Genet* **38**: 716-720.

**DeIulio GA, Guo L, Zhang Y, Goldberg JM, Kistler HC, Ma L-J. 2018.** Kinome Expansion in the Fusarium oxysporum Species Complex Driven by Accessory Chromosomes. *mSphere* **3**: 10.1128/msphere.00231-00218.

**Del Corpo D, Fullone MR, Miele R, Lafond M, Pontiggia D, Grisel S, Kieffer-Jaquinod S, Giardina T, Bellincampi D, Lionetti V. 2020.** AtPME17 is a functional Arabidopsis thaliana pectin methylesterase regulated by its PRO region that triggers PME activity in the resistance to Botrytis cinerea. *Molecular Plant Pathology* **21**: 1620-1633.

**Deslauriers SD, Larsen PB. 2010.** FERONIA Is a Key Modulator of Brassinosteroid and Ethylene Responsiveness in Arabidopsis Hypocotyls. *Molecular Plant* **3**: 626-640.

**Dobón A, Canet JV, García-Andrade J, Angulo C, Neumetzler L, Persson S, Vera P. 2015.** Novel Disease Susceptibility Factors for Fungal Necrotrophic Pathogens in Arabidopsis. *PLOS Pathogens* **11**: e1004800.

**Draeger C, Ndinyanka Fabrice T, Gineau E, Mouille G, Kuhn BM, Moller I, Abdou MT, Frey B, Pauly M, Bacic A, et al. 2015.** Arabidopsis leucine-rich repeat extensin (LRX) proteins modify cell wall composition and influence plant growth. *BMC Plant Biol* **15**: 155.

**Duan Q, Kita D, Li C, Cheung AY, Wu H-M. 2010.** FERONIA receptor-like kinase regulates RHO GTPase signaling of root hair development. *Proceedings of the National Academy of Sciences* **107**: 17821-17826.

**Fabrice TN, Vogler H, Draeger C, Munglani G, Gupta S, Herger AG, Knox P, Grossniklaus U, Ringli C. 2018.** LRX Proteins Play a Crucial Role in Pollen Grain and Pollen Tube Cell Wall Development. *Plant Physiol* **176**: 1981-1992.

**Guénin S, Mareck A, Rayon C, Lamour R, Assoumou Ndong Y, Domon J-M, Sénéchal F, Fournet F, Jamet E, Canut H, et al. 2011.** Identification of pectin methylesterase 3 as a basic pectin methylesterase isoform involved in adventitious rooting in Arabidopsis thaliana. *New Phytologist* **192**: 114-126.

**Herger A, Gupta S, Kadler G, Franck CM, Boisson-Dernier A, Ringli C. 2020.** Overlapping functions and protein-protein interactions of LRR-extensins in Arabidopsis. *PLOS Genetics* **16**: e1008847.

**Kadota Y, Sklenar J, Derbyshire P, Stransfeld L, Asai S, Ntoukakis V, Jones Jonathan D, Shirasu K, Menke F, Jones A, et al. 2014.** Direct Regulation of the NADPH Oxidase RBOHD by the PRR-Associated Kinase BIK1 during Plant Immunity. *Molecular Cell* **54**: 43-55.

**Kesten C, Gámez‐Arjona FM, Menna A, Scholl S, Dora S, Huerta AI, Huang HY, Tintor N, Kinoshita T, Rep M, et al. 2019.** Pathogen induced pH changes regulate the growth defense balance in plants. *The EMBO Journal* **38**: e101822.

**Lan Z, Song Z, Wang Z, Li L, Liu Y, Zhi S, Wang R, Wang J, Li Q, Bleckmann A, et al. 2023.** Antagonistic RALF peptides control an intergeneric hybridization barrier on Brassicaceae stigmas. *Cell* **186**: 4773-4787.e4712.

**Li C, Yeh F-L, Cheung AY, Duan Q, Kita D, Liu M-C, Maman J, Luu EJ, Wu BW, Gates L, et al. 2015.** Glycosylphosphatidylinositol-anchored proteins as chaperones and co-receptors for FERONIA receptor kinase signaling in Arabidopsis. *Elife* **4**: e06587.

**Manning VA, Pandelova I, Dhillon B, Wilhelm LJ, Goodwin SB, Berlin AM, Figueroa M, Freitag M, Hane JK, Henrissat B, et al. 2013.** Comparative Genomics of a Plant-Pathogenic Fungus, Pyrenophora tritici-repentis, Reveals Transduplication and the Impact of Repeat Elements on Pathogenicity and Population Divergence. *G3 Genes|Genomes|Genetics* **3**: 41-63.

**Merlot S, Leonhardt N, Fenzi F, Valon C, Costa M, Piette L, Vavasseur A, Genty B, Boivin K, Müller A, et al. 2007.** Constitutive activation of a plasma membrane H+ATPase prevents abscisic acid-mediated stomatal closure. *The EMBO Journal* **26**: 3216-3226.

**Müller MC, Praz CR, Sotiropoulos AG, Menardo F, Kunz L, Schudel S, Oberhänsli S, Poretti M, Wehrli A, Bourras S, et al. 2019.** A chromosome-scale genome assembly reveals a highly dynamic effector repertoire of wheat powdery mildew. *New Phytologist* **221**: 2176-2189.

**Peaucelle A, Louvet R, Johansen JN, Höfte H, Laufs P, Pelloux J, Mouille G. 2008.** Arabidopsis Phyllotaxis Is Controlled by the Methyl-Esterification Status of Cell-Wall Pectins. *Current Biology* **18**: 1943-1948.

**Schmid M, Davison TS, Henz SR, Pape UJ, Demar M, Vingron M, Schölkopf B, Weigel D, Lohmann JU. 2005.** A gene expression map of Arabidopsis thaliana development. *Nat Genet* **37**: 501-506.

**Shen Q, Bourdais G, Pan H, Robatzek S, Tang D. 2017.** *Arabidopsis* glycosylphosphatidylinositol-anchored protein LLG1 associates with and modulates FLS2 to regulate innate immunity. *Proceedings of the National Academy of Sciences* **114**: 5749-5754.

**Stegmann M, Monaghan J, Smakowska-Luzan E, Rovenich H, Lehner A, Holton N, Belkhadir Y, Zipfel C. 2017.** The receptor kinase FER is a RALF-regulated scaffold controlling plant immune signaling. *Science* **355**: 287-289.

**Wu Y, Ma X, Pan Z, Kale SD, Song Y, King H, Zhang Q, Presley C, Deng X, Wei C-I, et al. 2018.** Comparative genome analyses reveal sequence features reflecting distinct modes of host-adaptation between dicot and monocot powdery mildew. *BMC Genomics* **19**: 705.

**Xiao Y, Stegmann M, Han Z, DeFalco TA, Parys K, Xu L, Belkhadir Y, Zipfel C, Chai J. 2019.** Mechanisms of RALF peptide perception by a heterotypic receptor complex. *Nature* **572**: 270-274.

**Xu X, Chen X. 2023.** A complete genome sequence of Podosphaera xanthii race 2 F, the causal agent of powdery mildew isolated from cucumber in China. *Pathogens* **12**: 561.

**Zaccaron AZ, Neill T, Corcoran J, Mahaffee WF, Stergiopoulos I. 2023.** A chromosome-scale genome assembly of the grape powdery mildew pathogen *Erysiphe necator* reveals its genomic architecture and previously unknown features of its biology. *mBio* **14**: e00645-00623.
